# MATI, a Novel Protein Involved in the Regulation of Herbivore-Associated Signaling Pathways

**DOI:** 10.3389/fpls.2017.00975

**Published:** 2017-06-09

**Authors:** M. Estrella Santamaría, Manuel Martinez, Ana Arnaiz, Félix Ortego, Vojislava Grbic, Isabel Diaz

**Affiliations:** ^1^Centro de Biotecnología y Genómica de Plantas, Universidad Politécnica de Madrid – Instituto Nacional de Investigación y Tecnología Agraria y AlimentariaMadrid, Spain; ^2^Department of Biology, The University of Western Ontario, LondonON, Canada; ^3^Departamento de Biología Medioambiental, Centro de Investigaciones Biologicas, Consejo Superior de Investigaciones CientíficasMadrid, Spain

**Keywords:** plant–herbivore interaction, *Tetranychus urticae*, *Arabidopsis thaliana*, *Spodoptera exigua*, hormonal signaling pathways, plant redox status

## Abstract

The defense response of the plants against herbivores relies on a complex network of interconnected signaling pathways. In this work, we characterized a new key player in the response of Arabidopsis against the two-spotted spider mite *Tetranychus urticae*, the *MATI* (Mite Attack Triggered Immunity) gene. This gene was differentially induced in resistant Bla-2 strain relative to susceptible Kon Arabidopsis accessions after mite attack, suggesting a potential role in the control of spider mites. To study the *MATI* gene function, it has been performed a deep molecular characterization of the gene combined with feeding bioassays using modified Arabidopsis lines and phytophagous arthropods. The *MATI* gene belongs to a new gene family that had not been previously characterized. Biotic assays showed that it confers a high tolerance not only to *T. urticae*, but also to the chewing lepidopteran *Spodoptera exigua*. Biochemical analyses suggest that *MATI* encodes a protein involved in the accumulation of reducing agents upon herbivore attack to control plant redox homeostasis avoiding oxidative damage and cell death. Besides, molecular analyses demonstrated that *MATI* is involved in the modulation of different hormonal signaling pathways, affecting the expression of genes involved in biosynthesis and signaling of the jasmonic acid and salicylic acid hormones. The fact that *MATI* is also involved in defense through the modulation of the levels of photosynthetic pigments highlights the potential of MATI proteins to be exploited as biotechnological tools for pest control.

## Introduction

Plants are sessile organisms that rely on a battery of mechanisms to detect pathogens and pests in order to mount appropriate defense responses. Particularly, plants have evolved constitutive and inducible defenses to deter phytophagous arthropods, as well as indirect defenses, using volatiles and nectars to attract natural enemies of phytophagous insects and acari (Wu and Baldwin, 2010; Santamaria et al., 2013). As part of these defenses, herbivore-challenged plants can also emit volatiles to warn neighboring plants of an imminent threat (Muroi et al., 2011; Schuman and Baldwin, 2016). In parallel, herbivores respond to plant defenses by developing multiple strategies to avoid them (Elzinga and Jander, 2013; Suchan and Alvarez, 2015), and plants counterattack to implement emergency responses (Hao et al., 2008; Schmelz et al., 2012).

The induction of plant defenses is initiated when specific receptors recognize either the presence of herbivore (through the recognition of Herbivore-Associated Molecular Patterns; HAMPs), the damage incurred by plant tissues as a consequence of herbivore feeding (Damage-Associated Molecular Pattern; DAMPs) or the presence of volatiles emitted as plant–plant cues (Frost et al., 2007; Schuman and Baldwin, 2016). Recognition of these molecular patterns triggers transduction pathways that activate the expression of defense genes. Physiologically, early events in plant–herbivore interactions start with membrane potential depolarization at the feeding site, alteration in cell membrane and ion imbalance followed by changes in the intracellular Ca^2+^ and generation of reactive oxygen species (ROS) (Fürstenberg-Hägg et al., 2013). A cascade of protein kinases (CDPKs) as calcium-sensor proteins lead to the synthesis of phytohormones and activation of transcription factors that regulate the gene expression of a wide range of species-specific compounds with anti-nutritional, deterrent, repellent and toxic properties that function to entrap, inhibit, block or modify metabolism, development and fecundity of phytophagous arthropods (Wu and Baldwin, 2010; War et al., 2012; Santamaria et al., 2013). Host transcriptomic and proteomic profiles after arthropod feeding, oviposition or application of insect secretions have demonstrated that plants may discriminate between herbivores and activate specific plant responses (Giri et al., 2006; Kempema et al., 2007; Sotelo et al., 2014; Bandoly et al., 2015). Metabolomic approaches have corroborated plant’s ability to differentiate herbivore species and determine the onset of indirect defense responses to complement the direct defenses (Hettenhausen et al., 2013). Although the understanding of plant–arthropod interactions is still rudimentary, it is known that plant defenses locally or systemically induced by herbivores are regulated by a complex hormonal cross-talk (Erb et al., 2012; Wang and Wu, 2013; Gimenez-Ibanez et al., 2016). The central phytohormones that mediate between signal recognition and activation of defenses are Jasmonic Acid (JA), Salicylic Acid (SA), and Ethylene (Et). In general, JA regulates the induced defenses against chewing insects (Schmiesing et al., 2016), mesophyll sucking mites (Zhurov et al., 2014; Alba et al., 2015; Martel et al., 2015) and necrotrophic pathogens (Goossens et al., 2016), as well as mechanical wounding (Pieterse et al., 2009). SA-regulated responses are induced by phloem-feeding insects (Kawazu et al., 2012; Thaler et al., 2012), mesophyll sucking mites (Kant et al., 2004) and biotrophic pathogens (Gimenez-Ibanez et al., 2016), while Et most probably modulates the action of both hormones (Campos et al., 2014). Herbivory also leads to oxidative stress, changes in intracellular pH and desiccation, which modulate the JA pathway either directly or indirectly through the action of other hormones such as abscisic acid (ABA) (Hillwig et al., 2016). Other findings have also suggested important roles for auxins, cytokinins, and brassinosteroids (Diezel et al., 2009; Erb et al., 2012; Mur et al., 2013). However, a better understanding of phytohormone crosstalk transforming the initial perception events into appropriate responses is needed.

Phytophagous mites pierce parenchymatic plant cells using stylets to suck their nutrients, and cause severe chlorosis leading to a reduction in crop yield (Park and Lee, 2002; Farouk and Osman, 2011; Bensoussan et al., 2016). Among phytophagous mites, the two-spotted spider mite, *Tetranychus urticae*, is a species that feeds on more than 1,100 host plants including a wide range of ornamentals, greenhouse crops and annual and perennial field cultivars (Migeon and Dorkeld, 2006–2015). *T. urticae* is a model within chelicerate herbivores with its genome sequenced and with a broad range of tools and protocols developed (Grbic et al., 2011; Santamaria et al., 2012b; Cazaux et al., 2014). Mite ability to feed on *Arabidopsis thaliana* and the wide available toolkits for this plant species have provided an outstanding opportunity for functional studies of plant–mite interaction (Santamaria et al., 2012a, 2015; Zhurov et al., 2014). In addition, due to the spider mite impact in agriculture, research on plant responses to mite infestation has been also performed on crops (Maserti et al., 2011; Agut et al., 2015; Diaz-Riquelme et al., 2016). In tomato, the defense effect of glandular trichomes enriched in acylsugars or terpenoids against spider mites has been highlighted (Resende et al., 2002; Alba et al., 2009; Bleeker et al., 2012) as well as the repellence and oviposition deterrence induced by leaf extracts with high concentrations of methyl ketones (Antonious and Snyder, 2015). Tomato defenses against *T. urticae* also include the emission of different volatile compounds associated with the attraction of the spider mite predator *Phytoseiulus persimilis* (Ament et al., 2004). JA is essential but not the unique hormone for establishing the spider mite-induced defense responses (Kant et al., 2004; Ament et al., 2006; Zhurov et al., 2014; Martel et al., 2015). Spider mites feeding also triggers SA plant defense pathways (Kant et al., 2004; Zhurov et al., 2014). As a consequence, plants probably use this JA/SA balance to fine tune defense responses. However, spider mites may manipulate plant defenses downstream of hormonal crosstalk via an unknown mechanism generating considerable ecological costs (Kant et al., 2008; Glas et al., 2014; Alba et al., 2015; Wybouw et al., 2015). Recently, Villarroel et al. (2016) have identified effector/elicitor-like proteins in the mite saliva able to suppress plant defenses downstream of SA in tobacco and, as consequence, to promote mite’s reproductive performance. This salivary protein expression is dependent of the host plant (Jonckheere et al., 2016).

In this work, it is characterized the Arabidopsis *AT3G14395* gene of unknown function, termed *Mite Attack Triggered Immunity (MATI)*. The *MATI* gene was selected for further characterization because it showed consistently higher expression levels in the resistant Bla-2 strain relative to susceptible Kon Arabidopsis accessions after mite feeding. This previous result prompt us to study its potential defense properties as defense gene to control phytophagous arthropods. Here, we demonstrate that this gene encodes a novel small protein that contributes to confer plant protection against herbivores through modulation of plant redox status and hormonal signaling pathways.

## Materials and Methods

### Plant Material and Growth Conditions

*Arabidopsis thaliana* Col-0, Kondara (Kon), and Bla-2 (Bla-2) accessions (Nottingham Arabidopsis Seed Collection) were used as wild-types (WT). *A. thaliana* T-DNA mutants (SALK_092139C, N672907, referred as *mati* in this article) were obtained from the Arabidopsis Biological Resource Centre (ABRC^1^) through the European Arabidopsis Stock Centre (NASC^2^). T-DNA insertion and homozygous status and gene expression levels of the Salk lines were validated by conventional PCR and quantitative RT-qPCR assays (described below) (Supplementary Figure 1A). For growth in the soil, seeds were planted and incubated 5 days at 4°C. For *in vitro* growth, seeds were surface-sterilized with 75% (V/V) ethanol, dried, and plated onto Petri dishes containing 0.53 g of Murashige and Skoog salts (Sigma–Aldrich), 1% (W/V) sucrose, 0.5 g/L MES, and 0.4% (W/V) Phytagel (Sigma–Aldrich), adjusted to pH 5.7 with KOH. Plants and plates were then grown in growth chambers (Sanyo MLR-350-H) under control conditions (23°C ± 1°C, >70% relative humidity and a 16 h/8 h day/night photoperiod).

To generate overexpression lines, *MATI* cDNA from Col-0 plants was cloned into pGWB2 (CaMV35S, no tag) and pGWB5 (CaMV35S, C-sGFP) Gateway binary vectors (Nakagawa et al., 2007) using the specific primers included in Supplementary Table 1. The recombinant plasmids were introduced into *A. thaliana* Col-0 plants using *Agrobacterium* floral dip transformation (Clough and Bent, 1998), Col-*MATI* and Col-*MATI*-GFP plants in this article. pGWB2 plasmids were also introduced into *A. thaliana* Kon plants, termed Kon-*MATI* plants in this article. Shoots were regenerated on selective medium containing hygromycin (100 mg/L), and primary transformants (T0) were allowed to self-fertilize. Plants were then selected and self-fertilized twice more to generate the third generation lines (T3). Homozygous plants with one single copy insertion and the highest transgene expression levels coming from different transformation events were selected for our experiments (Supplementary Figure 1A).

### Gene Expression Analyses by Real Time PCR (RT-qPCR)

RT-qPCR assays have been used for different purposes: (i) to validate data from transcriptomic analysis; (ii) to determine the homozygous status of the Salk mutant lines; (iii) to study *MATI* gene expression in major Arabidopsis tissues. *A. thaliana* rosettes from Col-0, Bla-2, and Kon accessions were sampled after different time of mite infestation (1, 3, 6, 12, and 24 h) to validate microarray results. Total RNA was extracted following Oñate-Sánchez and Vicente-Carbajosa (2008) and reverse transcribed using Revert Aid^TM^ H Minus First Strand cDNA Synthesis Kit (Fermentas). cDNAs from *A. thaliana* Col-0 flowers, roots, siliques, leaves from stem rosettes at 1- to 3-week-old were also prepared. RT-qPCR was performed for three samples coming from three independent experiments as previously described (Santamaria et al., 2012a) using a SYBR Green Detection System (Roche) and the CFX Manager Software 2.0 (Bio-Rad). Gene expression was referred as relative expression levels (2^-ΔCt^) or fold change (2^-ΔΔCt^) (Livak and Schmittgen, 2001). Similarly, gene expression levels involved in hormonal signaling pathways were analyzed in rosettes from Col-*MATI*_4.1, -*mati* and WT plants in three samples coming from three independent experiments of non-infested 1 and 24 h infested plants (20 mites/plant). mRNA quantification was expressed as relative expression levels (2^-ΔCt^) normalized to ubiquitin (Livak and Schmittgen, 2001). Specific primers were designed through the Salk Institute T-DNA primer design link^3^ or through the PRIMER 3 program^4^. Primer sequences are indicated in Supplementary Table 1.

### Structural and Evolutionary *MATI* Gene Analyses

*Mite Attack Triggered Immunity* sequences were downloaded from the TAIR website^5^. Amino acid sequence was subjected to a sequence search in the Pfam database v28.0^6^ and SignalP 4.1 program^7^ to identify possible domains and a signal peptide within the protein, respectively. MATI proteins from different plant species were compiled using GreenphylDB v4^8^ and aligned by MUSCLE v3.8^9^. A phylogenetic tree was constructed by the maximum likelihood PhyML v3.0 method^10^, using a BIONJ starting tree and applying the approximate likelihood-ratio test (aLRT) as statistical test for non-parametric branch support. Displayed trees were visualized in the program MEGA 6.0^11^. Conserved sites were analyzed by drawing sequence logos representing profile hidden Markov models using the Skytign tool^12^. To analyze MATI gene product divergences among Arabidopsis accessions, its cDNA from Col-0, Bla-2, and Kon rosettes was cloned and sequenced using pGEM^®^-T Easy Vector Systems. The three sequences were aligned using Clustal W 1.83^13^.

### Subcellular Localization of MATI-GFP in Onion Epidermal Cells and *A. thaliana* Overexpression Lines

Mite Attack Triggered Immunity protein location within cell compartments were analyzed by two experimental approaches, using *MATI* gene fusions to the green fluorescent protein (GFP). First, transient expression assays were performed by particle bombardment of onion epidermal layers and second, stable transformation of Arabidopsis plants. The open reading frame (ORF) of the *MATI* gene translationally fused to the N-terminus of the whole reporter gene was cloned into the pGWB5 binary vector following Gateway technology instructions. As controls, the psmRS-GFP plasmid containing the cauliflower mosaic virus 35S promoter (Davis and Vierstra, 1998) and the pRTL2ΔNS/ss-RFP-HDEL plasmid containing the Arabidopsis chitinase signal sequence and the C-terminal HDEL ER retrieval signal, whose protein specifically localizes in the endoplasmic reticulum (ER) (Shockey et al., 2006), were used. Transient transformation of onion (*Allium cepa*) epidermal cells was performed by particle co-bombardment with a biolistic helium gun device (DuPont PDS-1000/Bio-Rad) as previously described (Diaz et al., 2005). Fluorescent images were acquired after 24 h of incubation at 22°C in the dark, under the LEICA SP8 confocal microscope (Leica). For the subcellular localization of the *MATI-GFP* fusion protein in *A. thaliana*, seedlings from Col-*MATI*_4.1-GFP line were observed under the LEICA SP8 confocal microscope (Leica) using the appropriated filters.

### Spider Mite and Beet Armyworm Maintenance and Fitness Analyses

To test the involvement of MATI in plant responses to herbivory*, T. urticae* and *Spodoptera exigua* feeding bioassays were performed in MATI overexpressing, *mati-*mutant ans Col-0 Arabidopsis lines. A colony of *T. urticae*, London strain (Acari: Tetranychidae) provided by Dr. Miodrag Grbic (UWO, Canada), was reared on beans (*Phaseolus vulgaris*) and maintained in growth chambers (Sanyo MLR-350-H) at 23°C ± 1°C, >70% relative humidity and a 16 h/8 h day/night photoperiod. Mites were synchronized by inoculating 100 adult females (random age) on one leaf of bean confined in a closed system under water-soaked cotton. After 1 day, adult females were removed, and 10 days after, population on the leaf was synchronized. Spider mite development and behavior was studied on Col-*MATI*_4.1, _5.2, *mati* and Col-0 WT plants. *T. urticae* fecundity assay was performed on detached leaves from 3-week-old plants. The newest emerged leaf (about 1 cm long) from each plant was fit in special dishes and infested with 12 adult synchronized females. After 36 h of infestation, the number of eggs was counted. Eight replicates were used for plant genotype.

*Spodoptera exigua* eggs were kindly provided by Drs. F. Budia and E. Viñuela (Department of Producción Agraria, ETSI Agronomos-UPM, Spain). Larvae were maintained in boxes feeding on artificial diet (Poitout and Bues, 1970), in growth chambers (Sanyo MLR-350-H) at 25°C ± 1°C, > 70% relative humidity and a 16 h/8 h day/night photoperiod. The boxes were lined with filter paper to reduce humidity. Vermiculite was provided for pupation when larvae complete its development cycle. Adults were allowed to emerge in cylindrical containers supplied with 10% (V/V) honey solution in water. Eggs were deposited on strips of filter paper and neonate (24 h) and L1 larvae (48 h) were synchronized for the plant and herbivore behavior assays, respectively. Larval performance was tested by placing three freshly hatched (neonate) beet armyworm larvae were placed on 3-week-old plants from the different genotypes (Col-*MATI*_4.1, -*mati* and WT lines) and larval weight was measured in a precision balance Mettler-Toledo MT5 (Mettler-Toledo) after 4 days of feeding. Twelve replicates were used for each genotype.

### Plant Damage Determination

Quantification of plant damage after arthropod feeding was done on Arabidopsis T2 entire plants from selected homozygous transgenic lines (Col-*MATI*_4.1, Col-*MATI*_5.2; Col-*mati*, Kon-*MATI*_6.1 and Kon-*MATI*_7.2) and from the non-transformed Col-0 and Kon controls. Three-week-old plants were infected with 20 *T. urticae* adults per plant. After 4 days of feeding, leaf damage was assessed by scanning the entire rosette using a hp scanjet (HP Scanjet 5590 Digital Flatbed Scanner series), according to Cazaux et al. (2014). Leaf damage was calculated in mm^2^, using Adobe Photoshop CS software. Six replicates were used for each genotype.

Leaf disks (7 mm diameter) of 3-week-old plants from the 3 Col-0 genotypes (WT, Col-*MATI*_4.1 and Col-*mati*) were infected with two neonate larvae of *S. exigua.* After 12 h, leaf disks were scanned using the hp scanjet (HP Scanjet 5590 Digital Flatbed Scanner series). Leaf damage was calculated in mm^2^ using split channel tool from imageJ software. Thirty two replicates were used for each genotype.

### Co-immunoprecipitation Assays

To identify MATI interaction partners, protein extracts from control and Col-*MATI*-GFP plants were immuno-purified using GFP-Trap system and analyzed in LTQ-Orbitrap Velos. Five gram of fresh weight of Col-GFP WT and Col-MATI-GFP (Col-MATI_4.1 line) plants grown for 3 weeks on half-strength MS medium were frozen in liquid nitrogen, extracted by grinding with a mortar and pestle, and added to 15 mL of cold TAP extraction buffer (50 mM TRIS-HCl, 150 mM ClNa, 10% (V/V) glycerol, 0.8% (V/V) Triton and 0.4% (W/V) Chaps (Sigma) and complete protease inhibitor cocktail (Roche). This lysate was centrifuged at 10,000 rpm for 10 min at 4°C and the protein content of the supernatant was quantified by Nanodrop^®^ND1000. Co-immunoprecipitation (Co-IP) experiments from Arabidopsis plants expressing GFP-tagged proteins were performed using GFP-Trap^®^ beads (ChromoTek GmbH), following the recommendations of the manufacturer. The immune-precipitated proteins were detected by immunoblot analysis on nitrocellulose membranes (GE Healthcare) using a monoclonal anti-GFP antibody (Miltenyi Biotec) at 1:5000 dilution in BSA blocking buffer containing 3% (WV) PBS and 0.05% (V/V) Tween-20. Acrylamide gels were staining with Coomassie and Oriole (Bio-Rad) to identify putative interactions.

### Proteomic Analysis

Co-immunoprecipitated proteins from Col WT (Col-0) and Col-*MATI*_4.1 samples were digested and analyzed in LTQ-Orbitrap Velos (Gil-Bona et al., 2015). Peptide identification from raw data was carried out using licensed version of search engine MASCOT 2.3.0 and Proteome Discoverer 1.4 (Thermo Scientific). Database search was performed against UniProt-Swissprot and NCBInr database with taxonomic restriction to *A. thaliana*. The following constraints were used for the searches: tryptic cleavage after Arg and Lys, up to two missed cleavage sites allowed, and tolerances of 20 ppm for precursor ions and 0.6 Da for MS/MS fragment ions. Besides, searches were performed allowing optional Methionine oxidation and fixed carbamidomethylation of Cysteine. Search against decoy database (integrated decoy approach) was used to FDR calculate and MASCOT percolator filter was applied to MASCOT results. The acceptance criteria for protein identification were a FDR < 1% and at least one peptide identified with high confidence (CI > 95%). Proteins identified as putative MATI interactors were functionally categorized using AgriGO v1.2^14^ and STRING v10^15^. In addition, the interaction among MATI putative partners was analyzed in STRING v10. The proteomic analysis was carried out at the Proteomics Facility UCM-PCM, a member of ProteoRed network.

### Electrolyte Leakage

Membrane depolarization is rapidly produced after plant–herbivore interaction followed by alterations in cell membrane and ion imbalance that can be monitored by measuring the electrolyte leakage (EL). Arabidopsis leaf disks (1 cm diameter) from Col-*MATI*_4.1, -*mati* and WT plants were infested with 10 mites and incubated for 24 h. The EL was determined as described by Masood et al. (2006). EL measurements were performed after 2 h (C1) of incubation at 32°C using a conductometer (EC-Metro BASIC 30, CRISON). Total electrolyte content was determined in the same way after boiling for 10 min (C2). Results were expressed as percentage of EL = (C1/C2) × 100. Six replicates were used for each genotype and treatment.

### Photosynthetic Pigment Measurements

To check whether plants reconfigure their primary metabolism during herbivore feeding to cope with the increased metabolic demands, chlorophyll and carotenoids were analyzed as standard parameters of photosynthetic activities in the three different Col-0 genotypes. Chlorophyll *a* and *b*, and total carotenoids were extracted from Col-*MATI*_4.1, -*mati*, WT lines. Arabidopsis rosettes were infested with 10 mites and incubated for 24 h. One hundred milligram of leaves were ground in a mortar with liquid nitrogen and suspended in 10 ml of 80% (V/V) acetone, using photo-protected tubes. After centrifugation at 3000 rpm for 15 min (L344 Eppendorf 5810R centrifuge), the absorbance of the supernatant was measured at 470, 663, and 646 nm, for carotenoids, chlorophyll *a* and *b*, respectively, using a UV-vis spectrophotometer (UltroSpec^TM^ 3300pro, Amersham Bioscience). Six replicates were used for each genotype and treatment. Pigment content was calculated using the extinction coefficients and equations determined by Lichtenthaler (1987).

### ROS Production and DAB Staining Quantification

The accumulation of H_2_O_2_ was determined using the 3,3-diaminobenzidine tetrachloridehydrate (DAB) substrate (Sigma–Aldrich) which produces a brown precipitate after oxidation in the presence of H_2_O_2_ (Martinez de Ilarduya et al., 2003). Col-0 Arabidopsis leaf disks (1 cm diameter) from Col-*MATI*_4.1, -*mati* and WT genotypes were infested with 10 mites and incubated for 24 h. Infested and non-infested control disks were stained with DAB following Rodríguez-Herva et al. (2012) and observed under a Zeiss Axiophot microscope. DAB staining specificity was confirmed in presence of the H_2_O_2_ scavenger, ascorbic acid (10 mM). ImageJ was used for the image quantification analysis^16^ using Methyl Green DAB vector. Six replicates were done by genotype and treatment.

### Thiol Quantification

The abundance of thiol groups was quantified in Col-*MATI*_4.1, -*mati* and WT a lines. Arabidopsis rosettes were infested with 10 mites and incubated for 24 h. Two hundred milligram of 3-week-old rosettes was homogenized with phosphate buffer (pH 6), centrifuged at 2500 rpm at 4°C for 10 min. Supernatant was used for the fluorometric thiol group assay following manufacturer instructions (Sigma–Aldrich). Six replicates were used for each genotype and treatment.

### Hormonal Analyses

Since hormones are important regulatory components of defense signaling, and particularly JA and SA are known to play major roles in regulating plant defense responses against spider mites, the accumulation of JA and SA as well as ABA in infested and non-infested Col-0 genotypes was also measured. Plant hormones (OPDA: 12-oxo-phytodienoic acid, JA, JA-Ile, SA, and ABA) were quantified by isotopic dilution mass spectrometry from 3-week-old rosettes of Col-*MATI*_4.1, -*mati* and WT plants after 24 h of spider mite feeding. Six rosettes were pooled per experiment and three independent experiments were performed. Isotope-labeled standards were added to plant samples (∼0.1 g) before extraction as described previously (Durgbanshi et al., 2005). Ultra-performance liquid chromatography (UPLC)-electrospray ionization-tandem mass spectrometry analyses were carried out on an Acquity SDS system (Waters) coupled to a triple quadrupole mass spectrometer (Micromass). Quantification was accomplished with an external calibration.

### Statistical Analysis

Statistical analyses were performed using one-way ANOVA for gene expression validation, expression in different tissues and damage analysis and *T. urticae* and *S. exigua* bioassays. Two-way ANOVA was used for gene expression validation, EL, H_2_O_2_, antioxidants, photosynthesis, metabolites and hormone gene expression studies. Student-Newman-Keuls multiple comparison test was applied to all the studies. In figures, significant differences (*P* < 0.05) among lines for different evaluated parameters, were reported with different letters. The *F*-value, *P*-value, and df (degree of fredom) obtained from each statistical analysis are compiled in Supplementary Table 2.

## Results

### *MATI*, a Gene Putatively Involved in Arabidopsis Defense against Spider Mites

**FIGURE 1:**
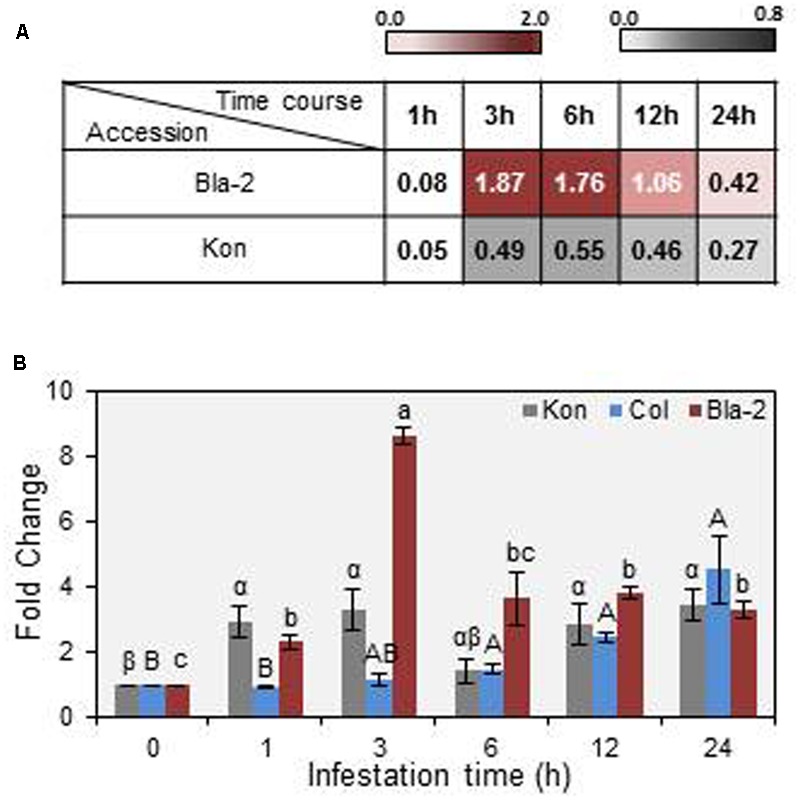
*Mite Attack Triggered Immunity* (*MATI*) (*AT3G14395)* gene expression in Arabidopsis Bla-2, Kon and Col-0 accessions in response to *Tetranychus urticae* infestation. **(A)** Microarray data from *MATI* expression in Arabidopsis Bla-2 and Kon accessions after *T. urticae* infestation at 1, 3, 6, 12, and 24 h post-infestation represented as Log(2). **(B)** Microarray validation of *MATI* gene expression after 1, 3, 6, 12, and 24 h post-infestation by RT-qPCR assays. Gene expression, referred as fold change (2^-ΔΔCt^). Data are means ± SE of three replicates. Different letters indicate significant differences between times post-infestation within each accession (*P* < 0.05, One-way ANOVA followed by Student-Newman-Keuls test).

**FIGURE 2:**
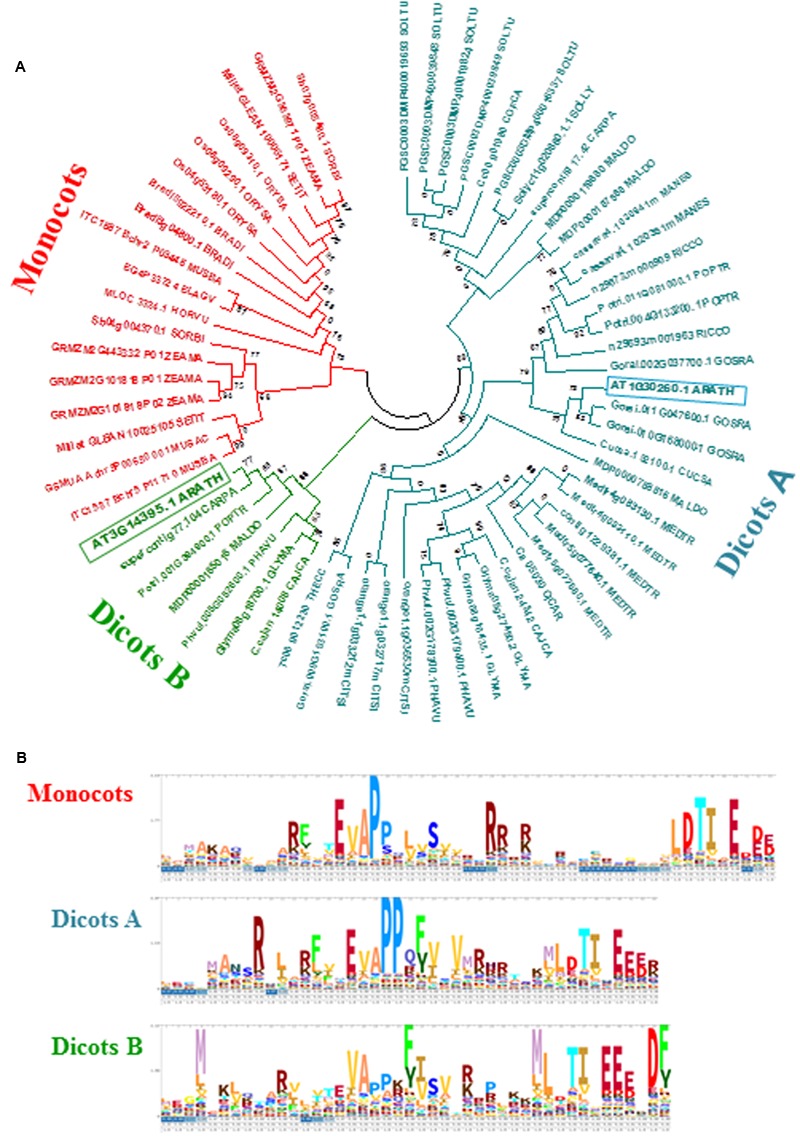
Evolutionary and sequence features of MATI proteins. **(A)** Phylogram of MATI homologs from selected plants. Phylogenetic tree constructed by the PhyML method using MATI homologs from 27 plant species. Numbers are aLRT values for statistical support. ARATH, *Arabidopsis thaliana*; BRADI, *Brachypodium distachyon*; CAJCA, *Cajanus cajan*; CARPA, *Carica papaya*; CICAR, *Cicer arietinum*; CITSI, *Citrus sinensis*; COFCA, *Coffea canephora*; CUCSA, *Cucumis sativus*; ELAGV, *Elaeis guineensis*; GLYMA, *Glycine max*; GOSRA, *Gossypium raimondii*; HORVU, *Hordeum vulgare*; MALDO, *Malus domestica*; MANES, *Manihot esculenta*; MEDTR, *Medicago truncatula*; MUSAC, *Musa acuminata*; MUSBA, *Musa balbisiana*; ORYSA, *Oryza sativa*; PHAVU, *Phaseolus vulgaris*; POPTR, *Populus trichocarpa*; RICCO, *Ricinus communis*; SETIT, *Setaria italica*; SOLLY, *Solanum lycopersicum*; SOLTU, *Solanum tuberosum*; SORBI, *Sorghum bicolor*; THECC, *Theobroma cacao*; ZEAMA, *Zea mays*. **(B)** Logos representing hidden Markov models of the N-terminal parts of the MATI domain for the three main groups identified.

Zhurov et al. (2014) highlighted the natural genetic variation of resistance between Arabidopsis accessions to *T. urticae*, identifying Bla-2 and Kon as the accessions at the opposing ends of the spectrum. Genome-wide analysis of transcriptional responses of these accessions demonstrated that they are very similar, which led to the conclusion that constitutive differences in levels of JA metabolites may underlay their differential resistance to mite feeding. Among otherwise similar responses, the *AT3G14395* gene showed consistently higher expression levels in the resistant Bla-2 strain relative to Kon (**Figure 1A**). These data were validated by RT-qPCR assays (**Figure 1B**). The *AT3G14395* gene, named *MATI*, encodes a specific plant protein of unknown function. The predicted protein, of 8.35 kDa, contains 75 amino acids with no known domains and no signal peptide. Searches in the GreenPhylDB discovered that homologs of the MATI protein were widely and uniquely present in angiosperms, including both monocots and dicots. A phylogenetic tree was constructed using protein sequences of MATI homologs from the 27 different species present in GreenPhylDB (**Figure 2A**). The phylogenetic analysis clearly identified three different groups, one formed by proteins from monocot species and two groups containing proteins from dicotyledonous plants (**Figure 2**). The MATI sequence was classified in the dicot group B together with other additional five protein sequences. Multiple sequence alignment revealed that *MATI* gene product was identical in Col-0, Bla-2, and Kon accessions (data not shown). A distant paralog, the *AT1G30260* from *A. thaliana* was present in the dicot group A (**Figure 2A**), but was not up-regulated after spider mite feeding in Bla-2 (Supplementary Figure 2).

Multiple sequence alignments of MATI homologs revealed the presence of a conserved N-terminal domain found in all protein sequences. A threonine-isoleucine pair followed by several negatively charged amino acid residues at the C-end of the conserved domain was invariably detected in all proteins. In addition, two proline residues toward the middle of the domain and several charged amino acids scattered along the conserved N-terminal domain were also identified in most cases (**Figure 2B**). Thus, *MATI* is a gene of an unknown function, but conserved among both mono- and di-cotyledon plants, potentially associated with plant responses to spider mite feeding.

### *MATI* Gene Expression and MATI Protein Subcellular Location

**FIGURE 3:**
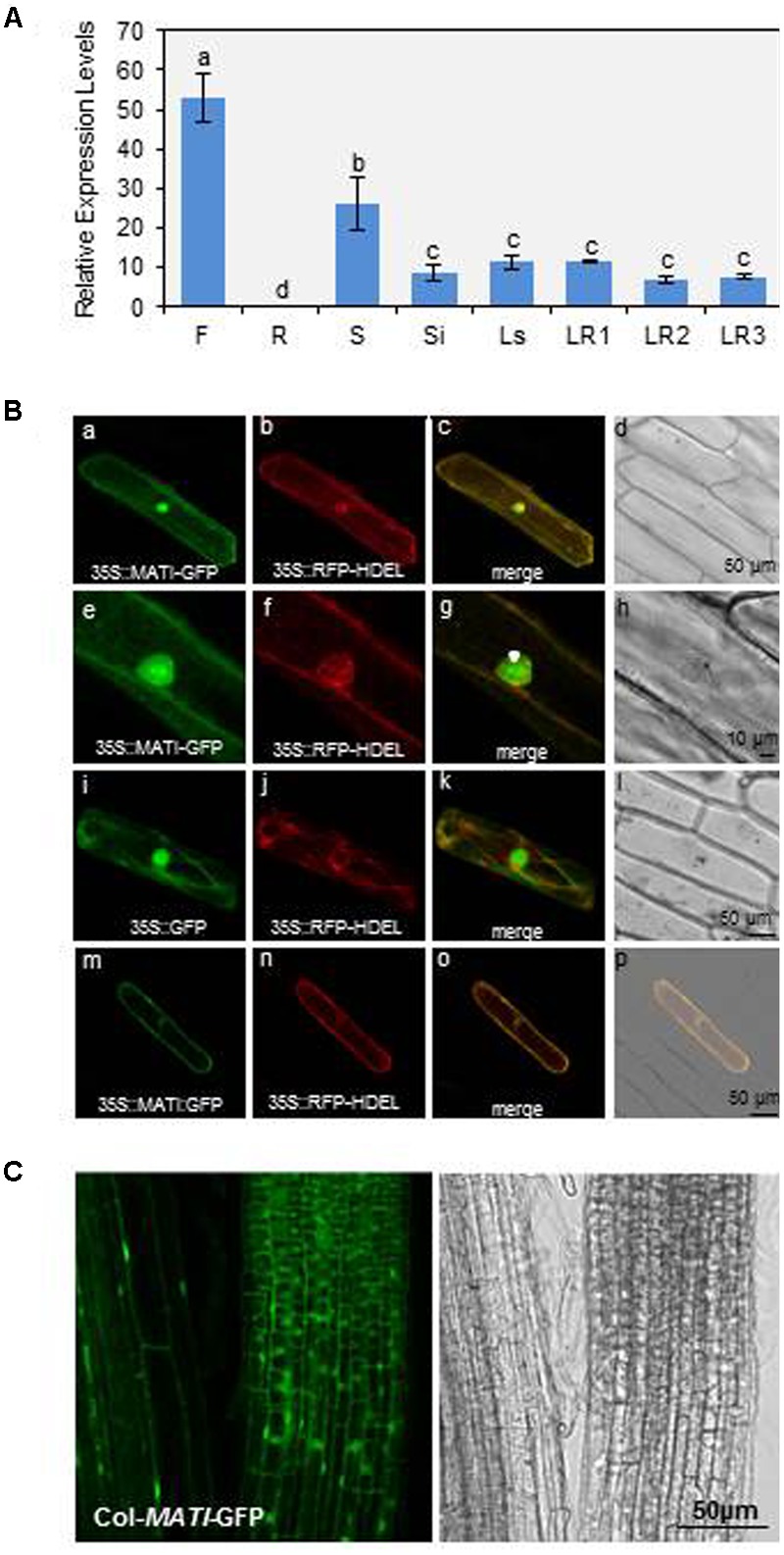
*Mite Attack Triggered Immunity* gene expression in plant tissues and protein subcellular location. **(A)** Relative gene expression levels (2^-ΔCt^) of *MATI* gene in different Arabidopsis tissues. Flowers (F), roots (R), seeds (S), siliques (Si), leaves from stem (Ls) and from rosettes at 1- to 3-week-old (LR1, LR2, and LR3). Data are means ± SE of three replicates. Different letters indicate significant differences (*P* < 0.05, One-way ANOVA followed by Student-Newman-Keuls test). **(B)** Confocal stacks spanning epidermal onion cells co-transformed with 35S-*MATI*-GFP, 35S-GFP control and/or 35S-RFP-HDEL control. Confocal projections from green fluorescent protein (GFP) **(a,e,i)**, RFP **(b,f,j)**, merged **(c,g,k)**, and the corresponding Nomarski snapshots **(d,h,l)**. Confocal images **(m,n)** and projections **(o,p)** of the MATI localization after plasmolysis with 1 M mannitol are shown. **(C)** Subcellular location of MATI protein in roots from transgenic Col-*MATI*-GFP lines. Observations with a Nomarski bright field are also shown (right panel). Bars are as indicated in all images.

To determine the expression of *MATI* gene, RT-qPCR studies were carried out in the major Arabidopsis tissues. *MATI* mRNA expression levels were mainly detected in flowers, seeds, siliques, and leaves from stem or rosette at different developmental stages, while it was scarcely detectable in roots (**Figure 3A**). Transient expression assays in onion epidermal cell layers were performed to determine MATI subcellular localization. The ORF of *MATI* translationally fused to the GFP was detected throughout the entire ER network, which was continuous with the nuclear membrane. This subcellular location was revealed by its co-location with the fluorescence emitted by 35S-RFP-HDEL plasmid, used as a control of endomembrane system location (**Figure 3Bb,f,j,n**). The MATI protein was also found in the plasma membrane, cytoplasm and within the nucleus (**Figure 3Ba,e**). Plasmolysis, induced by 1 M mannitol treatment confirmed the MATI location pattern in the cell-detached plasma membrane, with no fluorescence observed either in the cell wall or in the apoplast (**Figure 3Bm,o**). As expected, the 35S-GFP control showed fluorescence throughout the whole cell (**Figure 3Bi,k**). Furthermore, Col-*MATI*-GFP overexpressing lines were created to corroborate MATI subcellular location in the whole plant. The stable expression of 35S-*MATI*-GFP plasmid in transgenic Arabidopsis Col-0 plants confirmed the protein location in the endomembrane system, cytoplasm and nucleus as shown transgenic in roots of Arabidopsis transgenic plants (**Figure 3C**).

### Effects of MATI on Plant Resistance and Pest Performance

To investigate the role of MATI protein in plant defense, silenced lines (*mati* Salk) and overexpressing Arabidopsis Col-0 and Kon lines (Col-*MAT* and Kon-*MATI*, respectively) were generated. The characterization of the homozygous *mati* Salk line revealed a loss-of-function allele generated by the insertion of the T-DNA at the first part of the exon (Supplementary Figure 1A). On the other hand, gene expression analysis of transgenic plants expressing *MATI* gene constitutively in Col-0 and Kon backgrounds allowed the selection of lines that overexpressed the *MATI* ORF for further studies (Supplementary Figure 1Ba,b).

**FIGURE 4:**
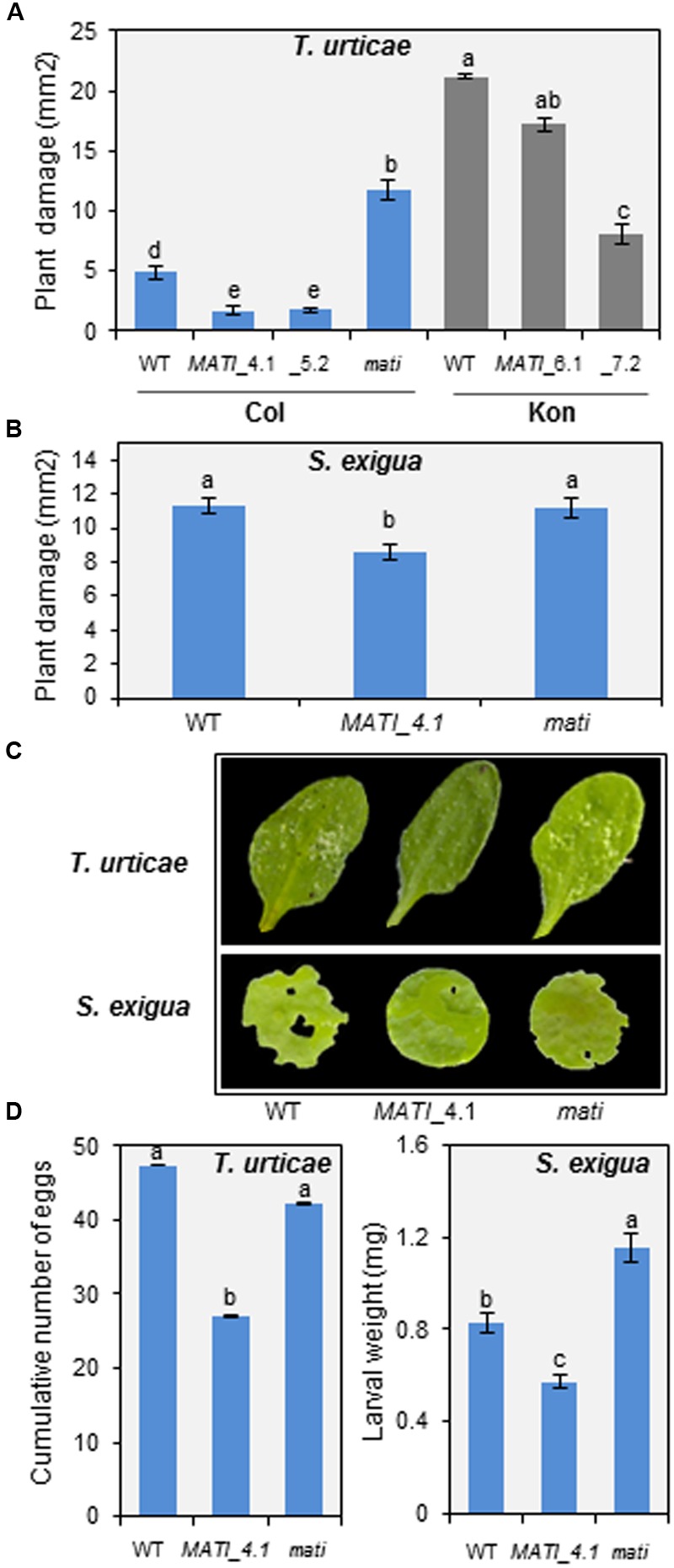
Plant damage of Col-0 and Kon genotypes infested with *T. urticae* and *Spodoptera exigua* and effects on pest perfomance. **(A)** Leaf damage on Arabidopsis Col-0 (WT, Col-*MATI*_4.1, _5.2, and Col-*mati* lines) and Kon (WT, Kon-*MATI*_6.1 and _7.2) genotypes 4 days after *T. urticae* feeding. **(B)** Leaf damage on Arabidopsis Col-0 (WT, Col-*MATI*_4.1, and Col-*mati* lines) 12 h after *S. exigua* feeding. Data are means ± SE of 6 (*T. urticae*) and 32 (*S. exigua*) replicates. Different letters indicate significant differences (*P* < 0.05, One-way ANOVA followed by Student-Newman-Keuls test). **(C)** Leaf phenotypes of Col-0 genotypes 4 days and 12 h after *T. urticae* and *S. exigua* feeding, respectively. **(D)** *T. urticae* and *S. exigua* performance, referred as number mite eggs and armyworm larval weight, after feeding for 4 days and 24 h, respectively, on Col-0 genotypes. Data are means ± SE 6 (*T. urticae)* and 32 (S. *exigua)* replicates. Different letters indicate significant differences (*P* < 0.05, One-way ANOVA followed by Student-Newman-Keuls test). Blue bars (Col background) and gray bars (Kon background).

Homozygous knock-out (Col-*mati*), overexpressing lines (Col-*MATI*_4.1, _5.2 and Kon-*MATI*_6.1, _7.2) as well as the corresponding WT plants were infested with spider mites and plant damage was quantified 4 days after mite feeding (**Figures 4A,C**). Col-*MATI* overexpressing lines showed approximately three times less damage than non-transformed Col WT infested plants (exactly 2.83- and 2.68-fold for Col-*MATI*_4.1 and _5.2, respectively). Similarly, overexpression of *MATI* in Kon (Kon-*MATI*_6.1 and _7.2) resulted in a significant decrease in damage (1.25- and 2.7-fold for Kon-*MATI*_6.1 and _7.2, respectively) in comparison to infested Kon WT. In contrast, damaged area in the knock-out Col-*mati* lines was about 2.5-fold greater than the damage quantified in Col-0 WT lines after mite feeding. The damage intensity measured as the chlorotic area on infested leaves was proportional to the *MATI* expression levels. To ensure that chlorotic area correlates with mite enhanced feeding and is not a consequence of greater cell death, mite performance were determined after feeding on plants with different levels of *MATI* expression. Fecundity assays were carried out on leaves from different Col-0 genotypes and showed that synchronized mites feeding on WT and *mati* plants had higher fecundity rates than the ones feeding on overexpressing *MATI* lines (**Figure 4D**). Thus, greater leaf damage reflects a mite’s ability to feed more intensely and to be more fecund, indicating that *MATI* overexpression correlates with plant’s capability to defend against herbivory.

To evaluate whether the plant defense mediated by *MATI* gene was specific to acari infestation, leaf disks from different Col-0 genotypes were infested with a generalist lepidopteran, the beet armyworm *S. exigua*, and the leaf damage was quantified. As shown in **Figures 4B,C**, the damaged area was significantly higher in Col-*mati* and Col-0 WT lines than in the overexpressing Col-*MATI*_4.1 line. In addition, the larval weight after 12 h of feeding on Col-*mati* and WT plants was twice and 1.5-fold greater, respectively, relative to the weight of the beet armyworm larvae fed on Col-*MATI*_4.1 (**Figure 4D**). Therefore, MATI overexpression confers plant protection against multiple herbivores.

### MATI Interactome Reveals Its Participation in a Complex Network

Using NCBInr database after Co-IP assays, 94 proteins were specifically found in *MATI* sample but not in the control WT. Most of these proteins were recognized by the identification of one peptide (Supplementary Table 3). The identified proteins were classified in five different over-represented categories based on their GO biological function related to sulfur compound metabolism, generation of precursor metabolites and energy, photosynthesis and Serine metabolism (Supplementary Figure 3A). When the identification was performed with a singular enrichment analysis in AgriGO, the Go biological process categories overrepresented included oxidation-reduction and metabolic processes, photosynthesis, and generation of precursor metabolites and energy (Supplementary Figure 3B). Additionally, the interactome network for the MATI putative partners revealed an enrichment of interactions between MATI putative interactors, with 247 observed interactions over the 100 expected for a total of 94 proteins. Among those partners putatively involved in sulfur compound metabolic process, 58 interactions were detected over the 6 expected (Supplementary Figure 3C). Since the two main overrepresented functional categories were related with sulfur metabolism and photosynthesis, different parameters involved in these physiological processes were further analyzed.

### *MATI* Modulates Plant Redox State and Photosynthesis

**FIGURE 5:**
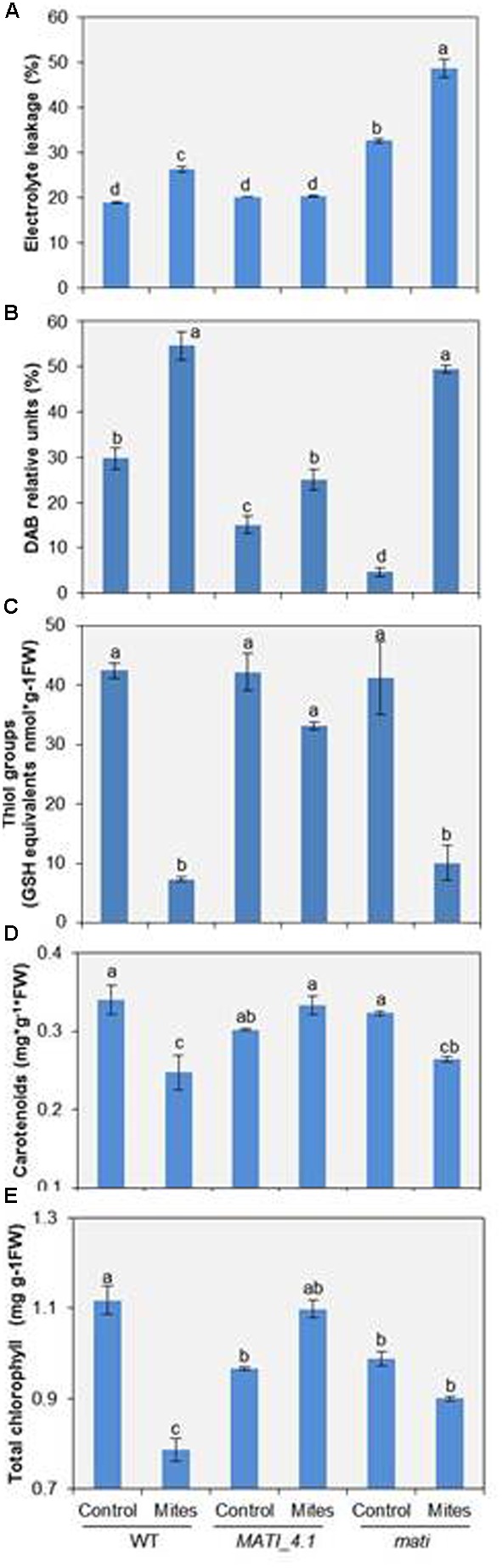
Redox status and photosynthetic pigment quantification in Arabidopsis Col-0 genotypes 24 h after *T. urticae* feeding. **(A)** Electrolyte leakage measurements. **(B)** Accumulation of hydrogen peroxide (DAB). **(C)** Total thiol groups. **(D)** Total carotenoids. **(E)** Total chlorophyll. WT, Col-*mati*, and Col-*MATI*_4.1 overexpressing lines were used. Data are means ± SE of six replicates. Different letters indicate significant differences (*P* < 0.05, Two-way ANOVA followed by Student-Newman-Keuls test).

Since *MATI* expression was induced early upon mite feeding on Arabidopsis leaves, it was important to determine how *MATI* might modulate plant responses to herbivore feeding. Thus, the EL was determined in Col-0 genotypes after mite feeding. Results showed a significant higher leakage either constitutively or mite-induced leakage in Col-*mati* plants than in Col-*MATI* overexpressing or in WT plants (**Figure 5A**). As these changes in conductance are usually accompanied by ROS generation, H_2_O_2_ concentrations were determined in the three Col-0 genotypes. The quantification of H_2_O_2_ in infested plants, expressed as DAB units, demonstrated that Col-0 WT and Col-*mati* plants accumulated about twice and ten-times more DAB, respectively, than their corresponding non-infested genotypes. However, the H_2_O_2_ levels in infested Col-*MATI_4.1* line changed only about 1.5-fold the DAB values relative to the non-infested line (**Figure 5B**). As thiol-type compounds are an important class of antioxidants that quench reactive oxidant species, variations in the total thiol group’s content among different Col-0 genotypes were also quantified. *T. urticae* feeding caused a significant decrease in the thiol’s group concentration in WT and Col-*mati* plants, while the thiol accumulation pattern was not significantly altered in Col-*MATI* samples (**Figure 5C**).

Three-week-old *mati*-mutant, overexpressing and control Col lines were used to determine chlorophyll and carotenoid content in the presence/absence of spider mites. While reduced levels of both pigments were observed in infested WT and Col-*mati* lines, high chlorophyll and carotenoid accumulation was detected in overexpressing Col-*MATI_4.1* lines after mite feeding in comparison to non-infested genotypes (**Figures 5D,E**). Consequently, *MATI* helps to maintain moderate levels of ROS necessary for defense signaling to control redox homeostasis, and to retain high levels of pigments to avoid photosynthetic cost.

### *MATI* Affects Plant Defense against Mites through Modulation of Hormonal Signaling

**FIGURE 6:**
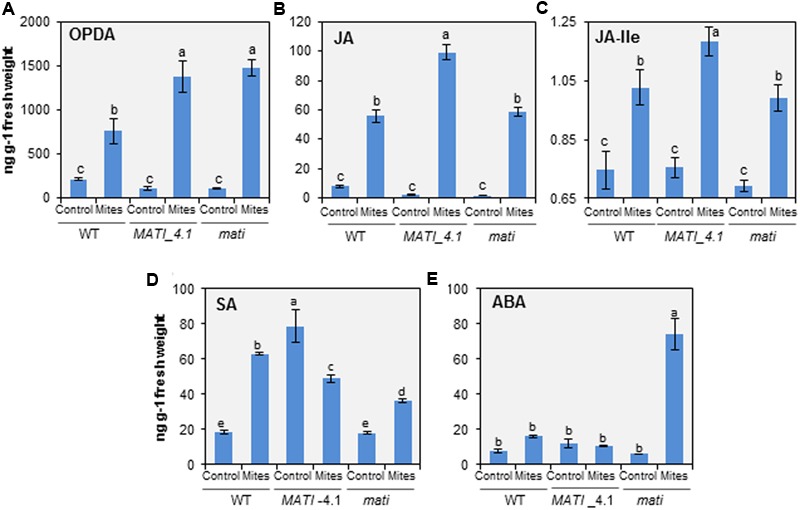
Quantification of hormones in Arabidopsis Col-0 genotypes infested with *T. urticae*. **(A)** OPDA. **(B)** Jasmonic Acid (JA). **(C)** JA-Ile. **(D)** Salicylic Acid (SA). **(E)** ABA. Levels were determined in 3-week-old rosettes from Col-*MATI*_4.1, Col-*mati*, and WT plants 24 h after spider mite feeding. Values, expressed in ng per g of fresh weight, are means ± SE of six replicates. Different letters indicate significant differences (*P* < 0.05, Two-way ANOVA followed by Student-Newman-Keuls test).

The hormonal analyses demonstrated that OPDA, JA, and its bioactive form JA-Ile, accumulated in leaf tissue during the initial 24 h in response to spider mite feeding, independently of the genotype (**Figures 6A–C**). The free OPDA content increased mainly in the spider mite-treated Col-*MATI* and -*mati* lines which contained about twice the JA precursor in infested WT (**Figure 6A**). The JA and JA-Ile accumulation also increased in Col-*mati* and WT genotypes, but the highest hormone levels were detected in the Col-*MATI* overexpressing lines after mite infestation. It was detected as an increase of approximately 45- and 16-fold in the JA and JA-Ile content, respectively (**Figures 6B,C**). Simultaneously, SA and ABA concentrations were also determined in the same rosette samples. Interestingly, high levels of SA were accumulated in non-infested Col-*MATI* lines but they were significantly reduced after mite feeding. In contrast, SA increased in response to infestation in Col-*mati* and WT lines (**Figure 6D**). Regarding ABA levels, they exclusively increased in infested Col-*mati* lines (**Figure 6E**). Thus, *MATI* is involved in the modulation of different hormonal signaling pathways in response to spider mite attack.

**FIGURE 7:**
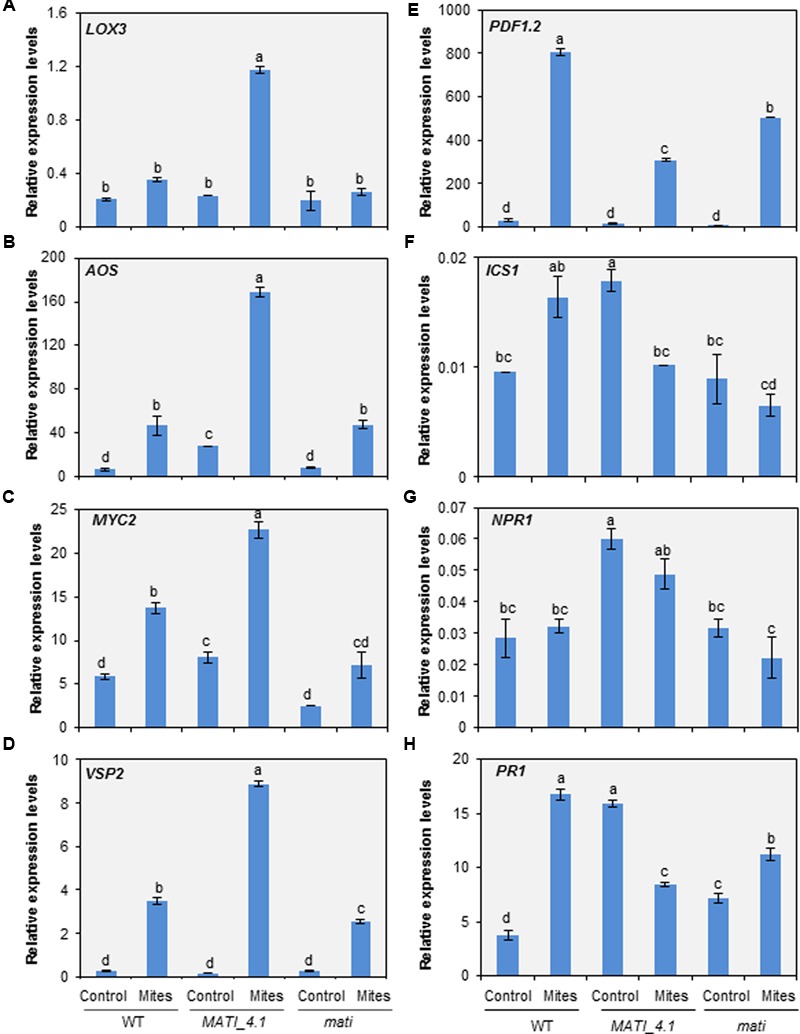
Expression levels of hormone-related genes in the three Col-0 genotypes. **(A)** Lipoxygenase 3 (*LOX3*). **(B)** Allene Oxide Synthase (*AOS*). **(C)** MYC2 transcription factor (*MYC2*). **(D)** Vegetative Storage Protein 2 (*VSP2*). **(E)** Plant Defensin (*PDF1.2*). **(F)** Isochorismate Synthase 1 (*ICS1*). **(G)** Non-expresser of PR gene1 (*NPR1*). **(H)** Pathogenesis Related (*PR1*). Genes were analyzed in Arabidopsis Col-*MATI*_4.1, Col-*mati*, and WT plants 24 h after mite feeding. Gene expression levels were normalized to the ubiquitin gene expression. Data are means ± SE of six replicates. Different letters indicate significant differences (*P* < 0.05, Two-way ANOVA followed by Student-Newman-Keuls test).

To further analyze the molecular basis of the *MATI* gene function, the expression pattern of some JA-, SA- and ABA-related genes located at different levels in the hormonal signaling pathways was analyzed 24 h after mite feeding. Lipoxygenase 3 (*LOX3*), Allene Oxide Synthase (*AOS*), MYC2 transcription factor (*MYC2*), vegetative storage protein 2 (*VSP2*), and plant defensin (*PDF1.2*) were the selected genes to dissect JA pathway. Non-infected Col WT plants showed low levels of constitutive expression of all JA-related genes but their expression increased after spider mite infestation. Interestingly, most of the selected defense genes presented higher mRNA levels in the infested overexpressing *MATI* than in WT or *mati* lines. Only the expression values of the *PDF1.2* gene related to the JA/Et defense branch were lower in Col-*MATI*_4.1 overexpressing line than in Col-*mati* or WT plants (**Figures 7A–E**). Similarly, Isochorismate Synthase 1 (*ICS1*), Non-expresser of PR gene1 (*NPR1*) and Pathogenesis Related (*PR1*) genes involved in SA-biosynthesis, regulation and final defense product, respectively, were selected to study SA implications. The three genes showed lower expression in infested than in non-infested Col-*MATI* lines while in Col-*mati* and WT lines the expression of some genes were increased, some decreased and other did not present differences in response to mites (**Figures 7F–H**). Additionally, the expression of the same JA- and SA-regulated genes was analyzed in early mite infestation assays, just 1 h after mite attack. Regarding the genes involved in the JA pathway, *LOX3, AOS*, and *MYC2* were up-regulated after mite feeding in WT, *MATI*, and *mati* lines while the *VSP2* gene was exclusively and highly induced in infested overexpressing *MATI* lines. Curiously, the *PDF1.2* gene was only induced in infested WT lines since its basal expression levels in non-infested *MATI* lines was as higher as the induced levels detected in WT plants after mite feeding. This gene was not induced in *mati* lines either infested or not infested (Supplementary Figures 4A–E). The expression of the genes involved in SA-biosynthesis, *ICS1, NPR1*, and *PR1*, was similar or lower in infested and non-infested Col-*MATI* lines but their expression levels in *mati* lines was not altered in response to mites. In contrast, *ICS1* and *NPR1* genes were up-regulated in infested WT lines and the *PR1* gene did not vary (Supplementary Figures 4F–H).

Besides, three genes related to ABA pathway, ABA deficient 1 (*ABA1*), Regulatory Component of ABA Receptor 3 (*RCAR3*), and Responsive to Dessication 22 (*RD22*), were also checked. The *ABA1* gene decreased after mite infestation following the same expression pattern in all genotypes, but *RCAR3* and *RD22* genes exclusively increased in *mati* knock-down lines after mite infestation (Supplementary Figure 5). All these results are in agreement with the hormonal content measured in the *MATI* overexpressing and knock-down genotypes and reinforce the importance of *MATI* in the regulation of different hormonal signaling pathways.

## Discussion

In the last years, many studies have been conducted to disclose the plant molecular events in response to phytophagous arthropods, which have revealed a complex combination of metabolic, developmental and signaling networks (Wu and Baldwin, 2010; War et al., 2012; Johnson et al., 2016; Schuman and Baldwin, 2016). In this scenario, the identification and further characterization of new molecules, compounds and pathways involved in the defense and the understanding of the mechanisms of plant protection is essential for pest control and crop improvement.

The *MATI* gene was selected as a potential candidate involved in defense against *T. urticae* based on its high expression levels in the resistant Bla-2 Arabidopsis accession after mite infestation in comparison to levels observed in the susceptible Kon accession (Zhurov et al., 2014). Our results confirm and support the wide role of the MATI protein in plant defense against herbivory based on: (i) the spider mite *T. urticae* inflicts more leaf damage in Col-*mati* knockdown than in Col-*MATI* overexpressing plants, and silencing of *MATI* improves mite performance; (ii) the Kon-*MATI* plants are able to partially revert the susceptible phenotype of Kon accession against spider mite attack and; (iii) larvae of the lepidopteran *S. exigua* grow more poorly on Col-*MATI* overexpressing lines and consumes less leaf tissue than do in knock-out *mati* or WT lines. Accordingly, previous transcriptomic studies showed the induction of MATI upon *Pieris rapae* herbivory (Coolen et al., 2016). MATI belongs to a protein family of unknown function highly conserved among angiosperm species, suggesting a potential role of this protein against herbivore attack across many species. Unfortunately, the absence of any domain of known function in its sequence made it difficult to decipher the molecular function of this small protein. To deal with its biological function, Co-IP assays were performed to identify putative protein interactors. It is assumed that proteins belonging to a biological complex should have related molecular functions, should be located in the same cellular compartment and should participate in a common physiological process (Zhang et al., 2008). Results revealed that MATI protein could be participating in a complex network since the classification of the putative interactors into biological categories highlighted related physiological processes, such as photosynthesis, oxidation-reduction pathways, redox homeostasis, sulfur metabolism and generation of precursor metabolites. Thus, MATI protective role seems to be involved in any aspect of the redox metabolism, and probably associated to buffering protein thiol groups against excessive oxidation. This putative physiological role can be easily integrated in the response of the plant to *T. urticae*.

In general, the initial plant–herbivore interaction triggers signal transduction pathways that commonly include Ca^2+^-signaling, production of ROS, phosphorylation cascades and transcriptional regulatory events leading to specific defense responses (Wu and Baldwin, 2010; War et al., 2012). Defensive responses begin when herbivores interact with the plant by introducing elicitors and triggering plant-derived signaling molecules (Zebelo and Maffei, 2015). The first consequence in the plant cell is the appearance of changes in ion fluxes, mainly Ca^2+^, associated to variations in the plasma membrane polarity leading to EL. Electrolyte leakage is almost instantaneously detected after stress application and it is usually accompanied by ROS accumulation (Demidchik et al., 2014). ROS are considered essential components in signaling toward pests, including spider mites (Leitner et al., 2005; Santamaria et al., 2012a). Excess of oxidative stress caused by H_2_O_2_ and strong plasma membrane depolarization finally result to programmed cell death. Conversely, moderate H_2_O_2_ concentrations and plasma membrane depolarization may differentially sense defense signaling (Foyer and Noctor, 2005; Bailey-Serres and Mittler, 2006; Baxter et al., 2014). Thiol groups are involved in reducing oxidative stress controlling the adverse effects on the cell survival exerted by ROS. Our findings indicate that after mite infestation leaves from Col-*mati* and WT plants showed a strong increase in the levels of H_2_O_2_ and electrolytic leakage together with a sharp decrease in the accumulation of thiol groups, probably associated to cell death promoted by acari feeding. In contrast, the infestation of *MATI* overexpressing plants do not lead to an increase of electrolytic leakage and causes a moderate accumulation of H_2_O_2_. Thus, the manipulation of plant antioxidant status should evidence a clear impact on herbivore performance and the subsequent plant defense phenotypes. According to this hypothesis, Arabidopsis *pad2-1* mutants (glutathione deficient) or *vtc1-1* mutants (ascorbic acid deficient) resulted more susceptible to *Spodoptera littoralis* than control lines (Schlaeppi et al., 2008). Thus, we can affirm that *MATI* gene is anyway involved in the regulation of the cell redox status mediated by H_2_O_2_ and thiol groups to protect the cell from an excessive oxidation and to facilitate the activation of defense signaling pathways.

Extensive characterization of plant–pest interactions has also demonstrated the integration between ROS and hormonal signaling in plant defense (Kerchev et al., 2012b; Mur et al., 2013). The feeding mode of the herbivore and the plant host determine the participation of phytohormones which appear to modulate the fine-tuning of defenses in response to each herbivore species. Generally, jasmonates are associated with the activation of defense responses toward a wide range of herbivorous insects (Howe and Jander, 2008; Wu and Baldwin, 2010). Likewise, JA-induction of plant defenses against spider mites has been widely described (Li et al., 2002, 2004; Ament et al., 2004; Schweighofer et al., 2007; Zheng et al., 2007; Zhang et al., 2009; Zhurov et al., 2014; Martel et al., 2015; Diaz-Riquelme et al., 2016). In Arabidopsis, Zhurov et al. (2014) reported the pivotal role of JA in establishing effective defenses against mites using mutants defective in JA biosynthesis (*aos*) and JA-regulated transcription (*myc*), which displayed increased susceptibility to *T. urticae* attack. As expected, we found that the biologically active jasmonate molecules JA and JA-Ile accumulated after 24 h mite infestation in all genotypes tested, but with a remarkably strong accumulation in Col-*MATI* overexpressing lines. These results link the redox balance to the presence of JA-regulated compounds and involve the *MATI* gene in the final modulation of both features. The question now is how *MATI* affects JA signaling pathway. In Arabidopsis, plant defenses against herbivores are differentially regulated by two different branches of the JA signaling pathway that are antagonistically controlled by the transcription factors MYC2 and ORA59. The specialist insect herbivore *P. rapae* induces the expression of *MYC2* and the MYC2-branch marker gene *VSP2*, and suppresses the transcription of *ORA59* and the JA/ethylene branch marker gene *PDF1.2* (Verhage et al., 2011; Vos et al., 2013). Our results show an overexpression of genes involved in the production of the JA precursor OPDA and in the JA-regulated responsive gene *VSP2*, concomitant with a minor induction of *PDF1.2* in the Col-*MATI* plants either at 1 h or at 24 h of infestation. These findings could be interpreted as a *MATI*-regulated preference of MYC2-branch against ORA59-branch. On the other hand, JA and JA-Ile production by Arabidopsis Col-0 plants also increased after the attack of the chewing insect *S. exigua* (Rehrig et al., 2014). Our findings with *S. exigua* suggest that the JA accumulation mediated by *MATI* gene also conferred resistance to this chewing insect extending its potential role as key defense modulator. These results correlate with the observed higher levels of JA and JA-Ile in overexpressing *MATI* plants, corroborating that JA is a functional output of the *MATI*-regulated Arabidopsis defense and suggesting that MATI exerts its function upstream in the MYC2-branch of the JA signaling pathway.

However, herbivore response does not imply the only activation of the JA signaling pathway. Other hormones are induced upon feeding in a specific plant–insect interaction (Nguyen et al., 2016) prompted by herbivore-specific associated molecular patterns (Acevedo et al., 2015; Xu et al., 2015). The extreme case is presented by many phloem-feeding insects, which mainly induce SA accumulation in the plant (Foyer et al., 2016). The fine-tuning of plant defense responses to specific attackers would be achieved by the cross-talk between JA and other phytohormones (Erb et al., 2012; Pieterse et al., 2012).

The antagonistic cross-talk effect between JA and SA signal transduction pathways have been explored at molecular level in the context of plant resistance to herbivores (Kawazu et al., 2012; Thaler et al., 2012; Vos et al., 2015). This antagonism has been exploited by herbivores to suppress JA-mediated defenses. For instance, the whitefly *Bemisia tabaci* induces the accumulation of SA in Arabidopsis, which plays a key role in the suppression of the effectual JA defenses of the host plant downstream JA signaling enhancing whitefly performance (Zhang et al., 2013). Despite this functional antagonism, there are several examples of plant–herbivore interactions leading to simultaneous JA and SA accumulation (Diezel et al., 2009; Chung et al., 2013; Zhang et al., 2013). SA content also increases in tomato and Arabidopsis in response to spider mite infestation (Kant et al., 2004; Glas et al., 2014; Zhurov et al., 2014). The significance of SA accumulation could depend on the specific plant–herbivore interaction, since profound divergences have been found in the transcriptional response of tomato and Arabidopsis plants to *T. urticae* (Zhurov et al., 2014; Martel et al., 2015) and in the tomato response to different *T. urticae* strains (Alba et al., 2015). In tomato, SA accumulation has been associated with a higher protective role against *T. urticae* (Kant et al., 2004, 2008; Villarroel et al., 2016). On the contrary, spider mite infested Arabidopsis plants deficient in SA biosynthesis or downstream signaling did not significantly alter plant damage or mite performance (Zhurov et al., 2014). Since SA induction could hamper JA-induced responses, the SA accumulation induced by spider mites in Arabidopsis could be a strategy to try to minimize accurate plant defense responses. Signaling effects caused by MATI are also involved in the modulation of SA levels. *MATI* overexpressing plants constitutively accumulate SA, which could be related to a higher basal resistance of these plants against pest/pathogens vulnerable to SA-induced defenses. After spider mite feeding, whereas in WT and Col-*mati* plants the SA accumulation strongly increase probably as a mechanism triggered by the mite to try to impede a correct defense response, the SA concentration in overexpressing MATI plants decreased, as a response leading to optimize the functionality of the JA-signaling pathway.

ABA signaling is associated to plant resistance to herbivores by interacting JA-signaling (Dinh et al., 2013; Vos et al., 2013). Commonly, this interaction promotes a higher protection but, Hillwig et al. (2016) demonstrated that ABA deficiency increases defense responses toward the aphid *Myzus persicae* which induces the SA signaling pathway. The ABA content increased in *mati* knock-down lines after mite infestation, but did not change in Col-*MATI* or WT plants. These results suggest a residual role of this hormone in the protective response against *T. urticae*. The effect on ABA accumulation in Col-*mati* plants could be due to a wide reprogramming of hormonal signaling pathways associated to the absence of the MATI protein.

Phytophagous arthropods have a clear impact on metabolism of the plant, limiting resources for growth and development in favor to plant defense (Huot et al., 2014). The classical plant response to herbivores involves photosynthesis reduction, stomata closure, enhanced respiration and changes in the cellular redox state, among other alterations (Kerchev et al., 2012a,b; Baxter et al., 2014). As expected, a decrease in pigment levels was found in infested Col-*mati* and WT lines associated to the damage observed in Arabidopsis leaves. In contrast, although leaves of Col-*MATI* lines were also damaged after mite feeding, they tended to accumulate chlorophyll and carotenoids. Interestingly, analysis of expression data for *MATI* gene in GENEVESTIGATOR^17^ evidences a strong silencing of this gene when plants are subjected to darkness treatments, linking MATI with the capacity of the cell to accumulate photosynthetic pigments. These results suggest that the cell reprogramming exerted by *MATI* is associated to a minimization of the trade-offs between plant defense and growth.

**FIGURE 8:**
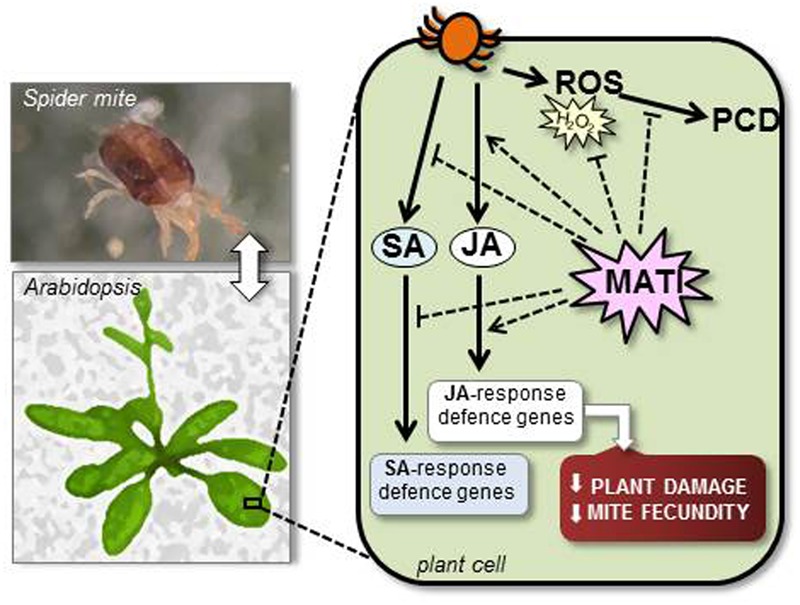
Proposed working model of MATI protein in plant defense against *T. urticae* attack. MATI protein enables the accumulation of antioxidants in response to spider mite feeding, controlling plant redox homeostasis to avoiding oxidative damage and cell death (PCD). Besides, MATI is involved in the modulation of the signaling pathways of JA and SA hormones, finally inducing JA effects. In consequence, MATI triggers plant tolerance to spider mites, reducing leaf damage and diminishing mite fecundity.

Taken all data together, a working model of MATI protein in defense against spider mites is proposed (**Figure 8**). Our findings suggest that *MATI* encodes a protein involved in the accumulation of reducing agents upon spider mite attack to control plant redox homeostasis avoiding excessive oxidative damage. Besides, MATI causes signaling effects that modulate different hormonal signaling pathways, affecting the expression of genes involved in biosynthesis and signaling of the JA and SA hormones. In consequence, MATI leads high plant tolerance to spider mites reducing leaf damage and mite fecundity. Future experiments will be performed to explain the exact role of MATI protein in this extremely coordinated modulation of signaling pathways and to analyze if MATI is also implicated in defense against other pests and pathogens. Furthermore, how MATI takes part in the modulation of the levels of photosynthetic pigments is a key question to address the potential of MATI proteins to be exploited as biotechnological tools.

## Author Contributions

ID, VG, and MM conceived the research. MS performed most of the experimental research. FO and AA participated in the insect feeding assays. ID, MM, VG, and MS participated in the design, the acquisition, analysis, or interpretation of data for the work. All authors contributed to final version of the manuscript.

## Conflict of Interest Statement

The authors declare that the research was conducted in the absence of any commercial or financial relationships that could be construed as a potential conflict of interest.

## Footnotes

1 https://abrc.osu.edu/

2 http://arabidopsis.info/

3 http://signal.salk.edu/tdnaprimers.2.html

4 http://bioinfo.ut.ee/primer3-0.4.0/

5 http://www.arabidopsis.org/

6 http://pfam.xfam.org/

7 http://www.cbs.dtu.dk/Services/SignalP/

8 http://www.greenphyl.org

9 http://www.ebi.ac.uk/Tools/msa/muscle

10 http://www.phylogeny.fr

11 http://www.megasoftware.net/

12 http://skylign.org/

13 http://www.ch.embnet.org/software/ClustalW.html

14 http://bioinfo.cau.edu.cn/agriGO/

15 http://string-db.org/

16 https://imagej.nih.gov/ij/

17 https://genevestigator.com/gv/
