# Supplementary material for: MATI, a Novel Protein Involved in the Regulation of Herbivore-Associated Signaling Pathways
Source: Front Plant Sci. 2017 Jun 9;8:975. doi: 10.3389/fpls.2017.00975 (PMC5466143; doi:10.3389/fpls.2017.00975)
Supplement: Supplementary file 1 [file Data_Sheet_1.pdf]

Supplementary Material

MATI, a novel Arabidopsis protein involved in the regulation of herbivore-associated signaling pathways

Santamaria ME, Martinez M, Arnaiz A, Ortego F, Grbic V, Diaz I*
*** Correspondence:** Isabel Diaz. E-mail: i.diaz@upm.es

1. Supplementary Figures and Tables

Supplementary Figure 1. Molecular characterization of *Arabidopsis* transgenic lines. (**A**) Col-*mati* line validation. (**a**) Scheme of the position of the T-DNA insertion (arrowhead) in the mutant Salk (SALK_092139C) *mati* line. (**b**) Location of the primers used for *mati* line validation and size of the expected products. See Supplementary Table 1 for the primers used. (**c**) PCR assays of *MATI* gene in Col-0 WT and *mati* lines to show homozygous status. Six day-old wild type and mutant seedlings were harvested and frozen in liquid nitrogen for DNA isolation (Lu et al., 2011). Four independent plants were analysed. **(d)** Relative expression levels of *MATI* gene normalized to UBQ in Col-WT and Col-*mati* plants 24h after mite feeding. (**B**) Expression levels of *MATI* gene in overexpressing Col-0 and Kon accessions (**a:**Col-*MATI*, **b:**Kon-*MATI* and **c:**Col-*MATI*-GFP lines) were determined by RT-qPCR assays. Gene expression, referred as fold change (2^{-ddCt}), was calculated normalizing the expression to the WT *MATI* gene. See Supplementary Table 1 for the primers used.

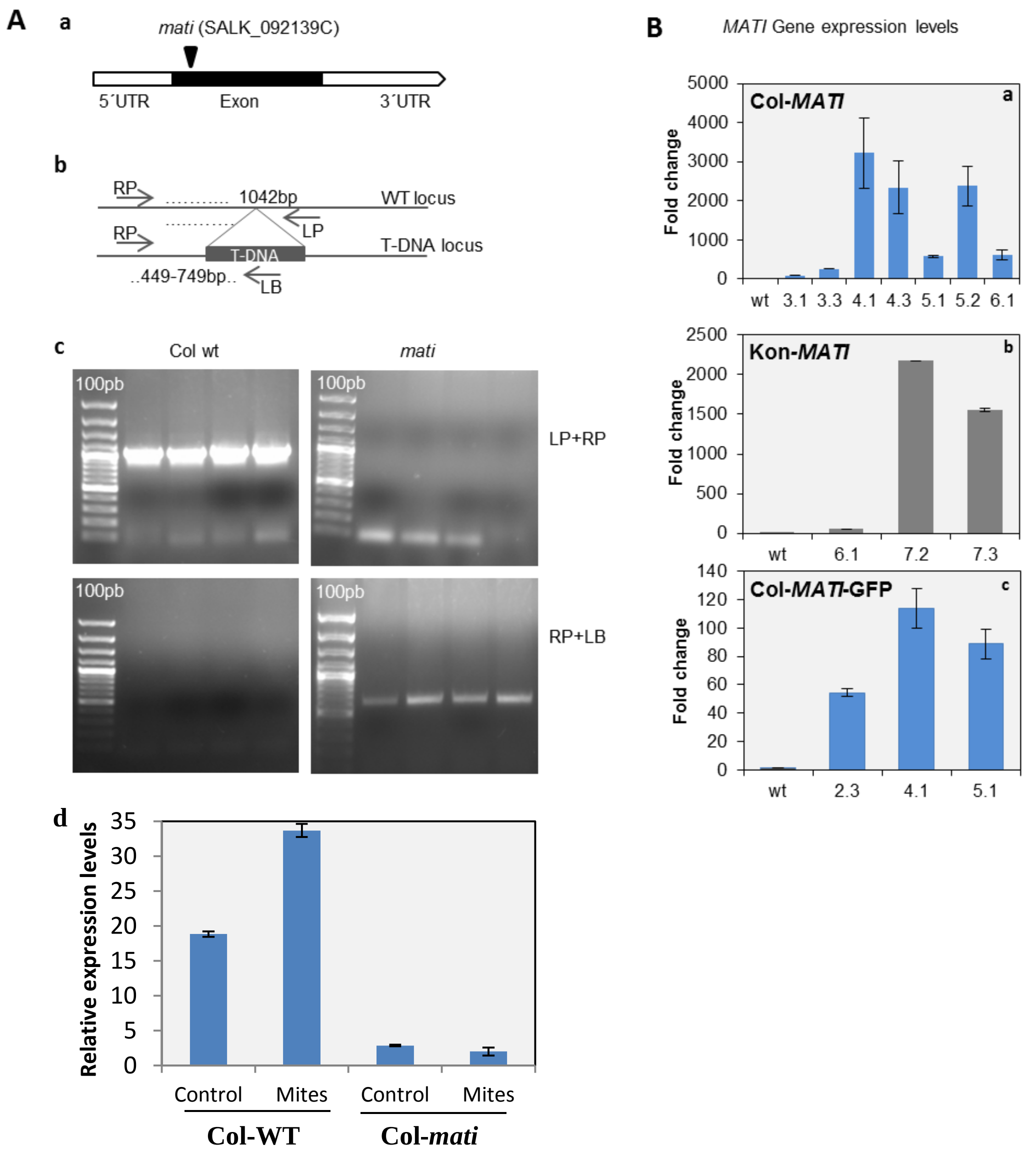

Supplementary Figure 2. Time course of *MATI* paralog (AT1G30260.1) gene expression in Bla-2 after spider mite infestation. Gene expression levels were normalized to the ubiquitin gene expression and to the expression in the uninfected plants. Primers were designed through PRIMER 3 (http://bioinfo.ut.ee/primer3-0.4.0/). See Supplementary Table 1 for the primers used.

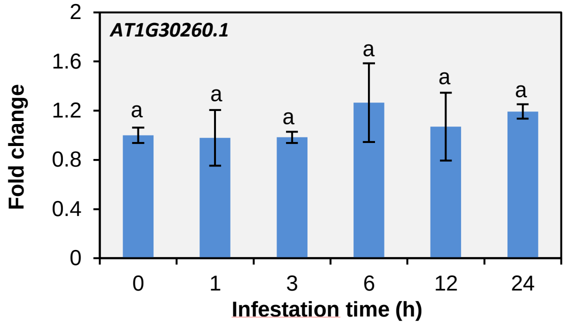

Supplementary Figure 3. Analysis of putative functional partners of MATI protein. **(A)** Functional enrichment classification of MATI putative interactors by GO Biological Process in STRING. **(B)** Singular Enrichment Analysis (SEA) in AgriGO. **(C)** Interactome of MATI partners removing disconnected nodes. Associations are represented from thick continuous lines for high score to thin discontinuous line for low score. Red balls represent proteins included in sulphur metabolism in STRING database.

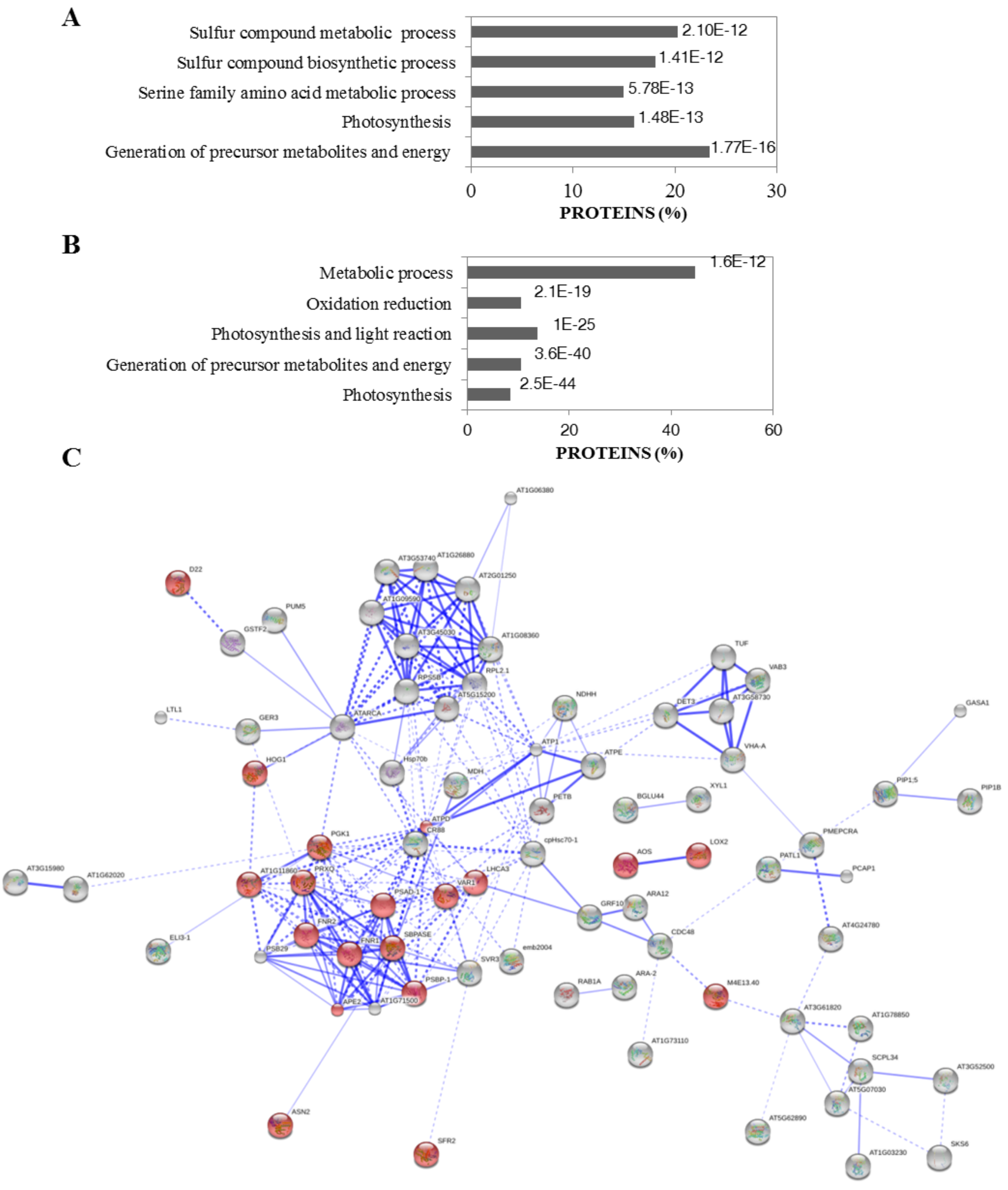

Supplementary Figure 4. Expression levels of hormone-related genes in the three Col-0 genotypes. **(A)** Lipoxygenase 3 (*LOX3*). **(B)** Allene Oxide Synthase (*AOS*). **(C)** MYC2 transcription factor (*MYC2*). **(D)** Vegetative Storage Protein 2 (*VSP2*). **(E)** Plant Defensin (*PDF1.2*). **(F)** Isochorismate Synthase 1 (*ICS1)*. **(G)** Non-expresser of PR gene1 (*NPR1)*. **(H)** Pathogenesis Related (*PR1).* Genes were analysed in Arabidopsis Col-*MATI*_4.1, *mati* and WT plants 1h after mite infestation. Gene expression levels were normalized to the ubiquitin gene expression. Data are means ± SE of three replicates. Different letters indicate significant differences (P <0.05, Two-way 1243 ANOVA followed by Student-Newman-Keuls test).

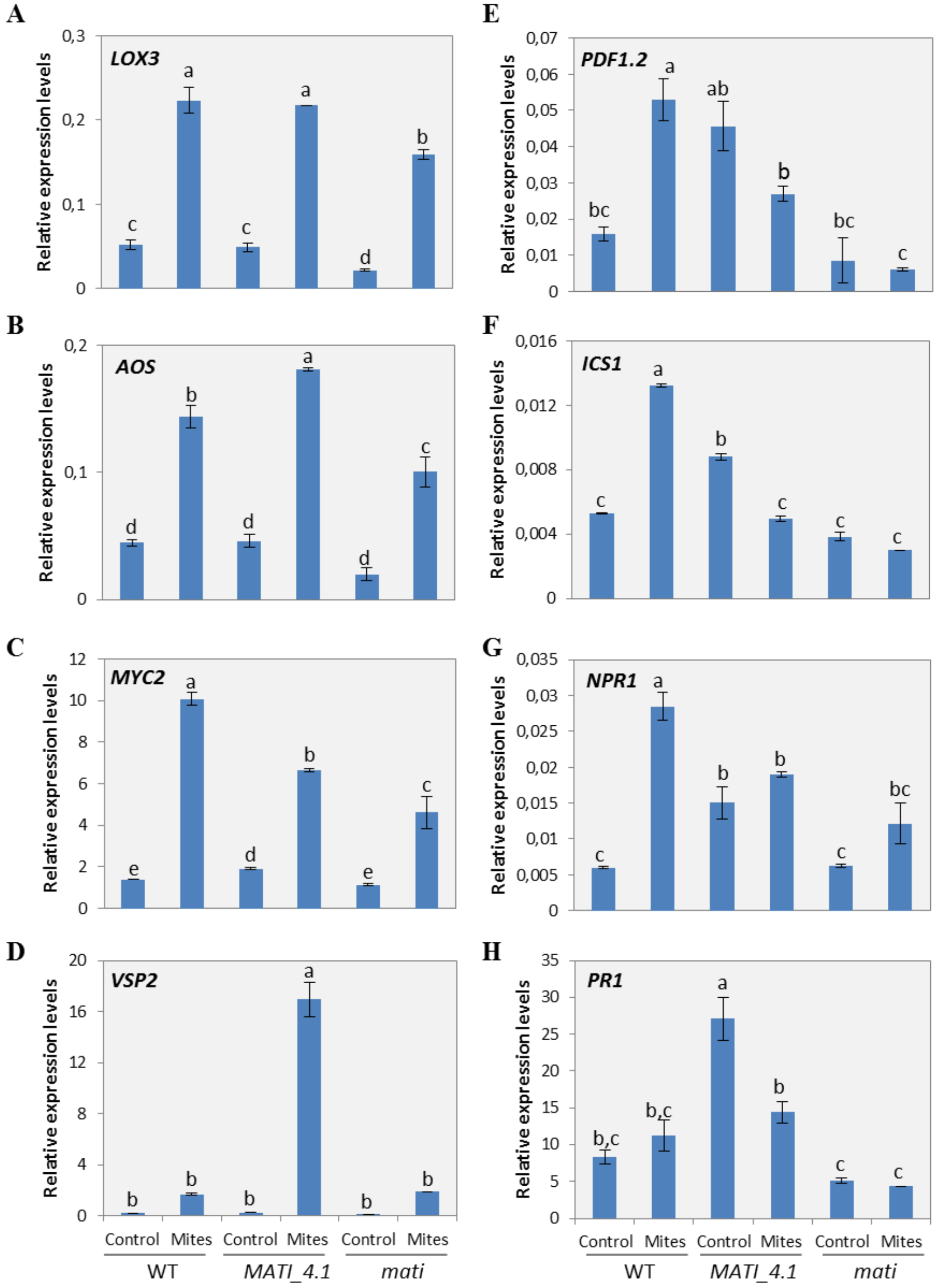

Supplementary Figure 5. Expression levels of ABA-related genes in the three Col-0 genotypes. **(A)** ABA deficient 1 (*ABA1*). **(B)** Regulatory Component of ABA Receptor 3 (*RCAR3*). **(C)** Responsive to Dessication 22 (*RD22*). Genes were analysed in Arabidopsis Col- *MATI*_4.1, Col-*mati* and WT plants after mite feeding. Gene expression levels were normalized to the ubiquitin gene expression and to the expression in the uninfected plants. Data are means ± SE of six replicates. Different letters indicate significant differences (P <0.05, Two-way ANOVA followed by Student-Newman-Keuls test).

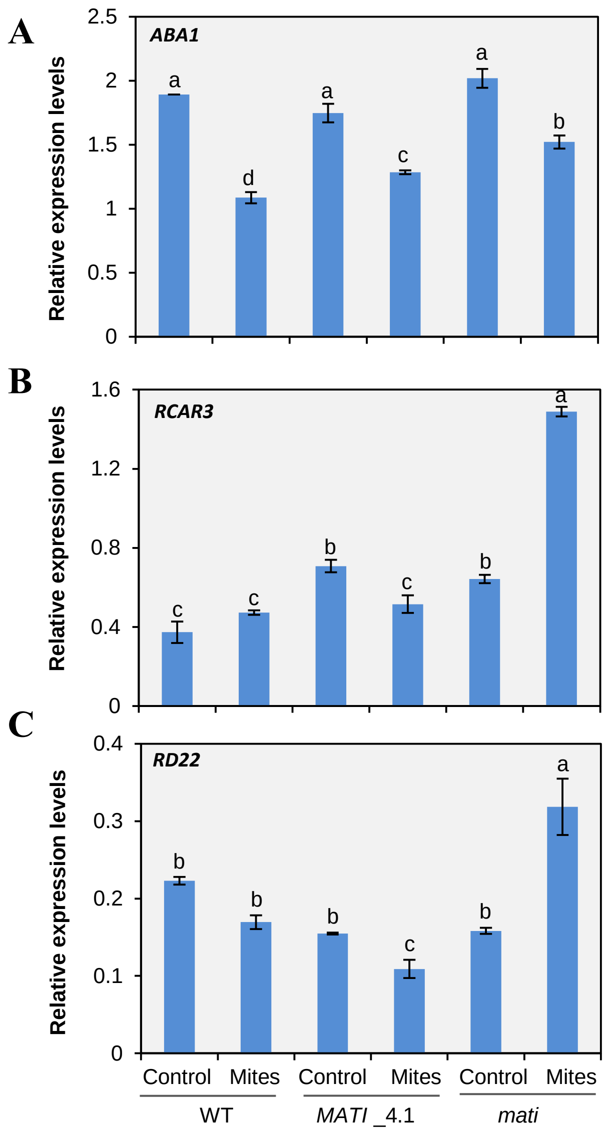

Supplementary Table 1. Sequences of oligonucleotide used primers (F: forward; R: reverse).

Gene	Primer name	Sequence (5´ → 3´)	Purpose
Ubiquitin	UBQ-F	GCTCTTATCAAAGGACCTTCGG	RT-qPCR-Gene expression assays
	UBQ-R	CGAACTTGAGGAGGTTGCAAAG	
AT3G14395	MATI-Seq-F	CCAACAATCTCTCATTTCTCT	PCR -Cloning for sequencing
	MATI-Seq-R	CTTCGCGTTGTTGAATGTTT	
AT3G14395	MATI-F	TGACAACAATCTCCGAGGAA	RT-qPCR-Gene expression assays
	MATI-R	GGCGTAGTGAGCAAGAGACC	
AT1G30260	MATI-Paralog-F	TCAGCTTCAGCTTCATGCAC	RT-qPCR-Gene expression assays
	MATI-Paralog-R	TTCTTGAAAACCGGAAAGGA	
T-DNA	LP	GTCGACTAGTGTTCGAGTGGG	PCR-T-DNA insertion validation
	RP	TTTGGTGACTCGTGCATAGTG	
	BP643	GCAATCAGCTGTTGCCCGTCTCACTGGTG	
AT3G14395	pGWB:MATI-F	CACCATGTCGGGAGCATCTTCT	PCR-Cloning
	pGWB2:MATI-R	TTAGTATTTGGGACGAATTGTA	
	pGWB5:MATI-R	AAAGTATTTGGGACGAATTGTA	
AT5G42650	AOS-F	CGTTAGGAAGCTCCGTTAATTTCTC	RT-qPCR-Gene expression assays
	AOS-R	TTCACGAAACTGGAACAAGAAAACA	
AT1G32640	MYC2-F	TCCGAGTCCGGTTCATTCT	
	MYC2-R	TCTCGGGAGAAAGTGTTATTGAA	
AT1G17420	LOX3-F	CGCCAATCAACAGTTTCTGA	
	LOX3-R	CTCGTCTCGTGGCACATACA	
AT5G24770	VPS2-F	ATGCCAAAGGACTTGCCCTA	
	VPS2-R	CGGGTCGGTCTTCTCTGTTC	
AT5G44420	PDF1.2-F	GTTCTCTTTGCTGCTTTCGAC	
	PDF1.2-R	GCAAACCCCTGACCATGT	
AT1G74710	ICS1-F	CAAATCTCAACCTCCGTCGT	
	ICS1-R	AATCAATTGCTCCGATTTGC	
AT1G64280	NPR1-F	TGCATCAGAAGCAACTTTGG	
	NPR1-R	GGCCTTTGAGAGAATGCTTG	
AT2G14610	PR1-F	TCAGTGAGACTCGGATGTGC	
	PR1-R	CGTTCACATAATTCCCACGA	
AT5G67030	ABA1-F	CTCACTGACAAAGCCGATGA	
	ABA1-R	GTCGCCGTGTGGAATTAGAT	
AT5G53160	RCAR3-F	TGTGGTGAAAGGAAACATGG	
	RCAR3-R	TCACCACCAACGATTCTGAT	
AT5G25610	RD22-F	CCGGTAAAAGAACCGACGTA	
	RD22-R	ATCGTCGTGGAGCTGAGTCT	

Supplementary Table 2. Statistical results obtained with GraphPad Prism 6 sofware. The F-value, the degrees of freedom from the numerator (dfn) and denominator (dfd) and the P-value are indicated. For the ANOVA two factor tests the F-value is indicated for the interaction (I), the treatment (T) and the MATI genotype (G).

Figure	Test	$F_{(dfn,dfd)}$	P
1B	ANOVA One Factor (Kon)	$F_{(5,12)}$=60.10	P<0.0001
	ANOVA One Factor (Col)	$F_{(5,12)}$=27.90	P<0.0001
	ANOVA One Factor (Bla-2)	$F_{(5,12)}$=289.90	P<0.0001
3A	ANOVA One Factor	$F_{(7,16)}$=23.83	P<0.0001
4A	ANOVA One Factor	$F_{(6,35)}$=73.88	P<0.0001
4B	ANOVA One Factor	$F_{(2,93)}$=10.73	P<0.0001
4D	ANOVA One Factor (*T.urticae*)	$F_{(2,21)}$=9.48	P=0.0012
	ANOVA One Factor (*S.exigua*)	$F_{(2,98)}$=42.26	P<0.0001
5A	ANOVA Two Factor	$F_{(2,30)}$=42.60 (I)<br>$F_{(1,30)}$ =124.6 (T)<br>$F_{(2,30)}$=332.9 (G)	P<0.0001<br>P<0.0001<br>P<0.0001
5B	ANOVA Two Factor	$F_{(2,30)}$=46.15(I)<br>$F_{(1,30)}$=318 (T)<br>$F_{(2,30)}$=77.54 (G)	P<0.0001<br>P<0.0001<br>P<0.0001
5C	ANOVA Two Factor	$F_{(2,30)}$=35.64 (I)<br>$F_{(1,30)}$=186 (T)<br>$F_{(2,30)}$=37.11(G)	P<0.0001<br>P<0.0001<br>P<0.0001
5D	ANOVA Two Factor	$F_{(2,30)}$=10.41 (I)<br>$F_{(1,30)}$=9.97 (T)<br>$F_{(2,30)}$=1.749 (G)	P<0.0001<br>P<0.0001<br>P<0.0001
5E	ANOVA Two Factor	$F_{(2,30)}$=26.09 (I)<br>$F_{(1,30)}$=13.41 (T)<br>$F_{(2,30)}$=4.6 (G)	P<0.0001<br>P=0.001<br>P=0.0177
6A	ANOVA Two Factor	$F_{(2,30)}$=10.37 (I)<br>$F_{(1,30)=}$171.9 (T)<br>$F_{(2,30)}$=5.509 (G)	P<0.0001<br>P<0.0001<br>P<0.0001
6B	ANOVA Two Factor	$F_{(2,30)}$=39.48 (I)<br>$F_{(1,30)=}$777.0 (T)<br>$F_{(2,30)}$=29.93 (G)	P<0.0001<br>P<0.0001<br>P<0.0001
6C	ANOVA Two Factor	$F_{(2,30)}$=1.632 (I)<br>$F_{(1,30)}$=67.19 (T)<br>$F_{(2,30)}$=3.265 (G)	P=0.2715<br>P<0.0001<br>P=0.0521
6D	ANOVA Two Factor	$F_{(2,30)}$=45.92 (I)<br>$F_{(1,30)}$=11.17 (T)<br>$F_{(2,30)}$=44.39 (G)	P<0.0001<br>P=0.0018<br>P<0.0001
6E	ANOVA Two Factor	$F_{(2,30)}$=48.61 (I)<br>$F_{(1,30)}$=63.79 (T)<br>$F_{(2,30)}$=36.77 (G)	P<0.0001<br>P<0.0001<br>P<0.0001
7A	ANOVA Two Factor	$F_{(2, 12)}$=91.91 (I)<br>$F_{(1, 12)}$=174.3 (T)<br>$F_{(2, 12)}$=108.1 (G)	P < 0.0001<br>P < 0.0001<br>P < 0.0001
7B	ANOVA Two Factor	$F_{(2, 12)}$=266.9 (I)<br>$F_{(1, 12)}$=1270 (T)<br>$F_{(2, 12)}$=532.2 (G)	P < 0.0001<br>P < 0.0001<br>P < 0.0001
7C	ANOVA Two Factor	$F_{(2, 12)}$=56.52 (I)<br>$F_{(1, 12)}$=541.0 (T)<br>$F_{(2, 12)}$=245.0 (G)	P < 0.0001<br>P < 0.0001<br>P < 0.0001
7D	ANOVA Two Factor	$F_{(2, 12)}$=1768 (I)<br>$F_{(1, 12)}$=9676 (T)	P < 0.0001<br>P < 0.0001

		$F_{(2, 12)}$=1637 (G)	P < 0.0001
7E	ANOVA Two Factor	$F_{(2, 12)}$=1233(I)<br>$F_{(1, 12)}$=17317 (T)<br>$F_{(2, 12)}$=1412 (G)	P < 0.0001<br>P < 0.0001<br>P < 0.0001
7F	ANOVA Two Factor	$F_{(2, 12)}$=7.634 (I)<br>$F_{(1, 12)}$=0.5050 (T)<br>$F_{(2, 12)}$=6.408 (G)	P = 0.0073<br>P = 0.4909<br>P = 0.0128
7G	ANOVA Two Factor	$F_{(2, 12)}$=1.374 (I)<br>$F_{(1, 12)}$=1.969 (T)<br>$F_{(2, 12)}$=17.71 (G)	P = 0.2902<br>P = 0.1859<br>P = 0.0003
7H	ANOVA Two Factor	$F_{(2, 12)}$=229.1 (I)<br>$F_{(1, 12)}$=66.38 (T)<br>$F_{(2, 12)}$=20.29 (G)	P < 0.0001<br>P < 0.0001<br>P = 0.0001
S2	ANOVA One factor	$F_{(5, 12)}$=0.3708	P = 0.8591
S4A	ANOVA Two Factor	$F_{(2, 12)}$=9.653 (I)<br>$F_{(1, 12)}$ = 2035 (T)<br>$F_{(2, 12)}$=72.55 (G)	P = 0.0032<br>P < 0.0001<br>P < 0.0001
S4B	ANOVA Two Factor	$F_{(2, 12)}$=6.433 (I)<br>$F_{(1, 12)}$=225.9 (T)<br>$F_{(2, 12)}$=17.23 (G)	P = 0.0126<br>P < 0.0001<br>P = 0.0003
S4C	ANOVA Two Factor	$F_{(2, 12)}$ = 99.30 (I)<br>$F_{(1, 12)}$ = 1232 (T)<br>$F_{(2, 12)}$ = 98.57 (G)	P < 0.0001<br>P < 0.0001<br>P < 0.0001
S4D	ANOVA Two Factor	$F_{(2, 12)}$=85.73 (I)<br>$F_{(1, 12)}$=159.3 (T)<br>$F_{(2, 12)}$=87.88 (G)	P < 0.0001<br>P < 0.0001<br>P < 0.0001
S4E	ANOVA Two Factor	$F_{(2, 12)}$=12.34 (I)<br>$F_{(1, 12)}$=1.413 (T)<br>$F_{(2, 12)}$=14.15 (G)	P = 0.0012<br>P = 0.2576<br>P = 0.0007
S4F	ANOVA Two Factor	$F_{(2, 12)}$=81.56 (I)<br>$F_{(1, 12)}$=7.71 (T)<br>$F_{(2, 12)}$=75.03 (G)	P < 0.0001<br>P = 0.0168<br>P < 0.0001
S4G	ANOVA Two Factor	$F_{(2, 12)}$=19.42 (I)<br>$F_{(1, 12)}$=66.02 (T)<br>$F_{(2, 12)}$=15.90 (G)	P < 0.0001<br>P < 0.0001<br>P < 0.0001
S4H	ANOVA Two Factor	$F_{(2, 12)}$=11.65 (I)<br>$F_{(1, 12)}$=7.909 (T)<br>$F_{(2, 12)}$=49.20 (G)	P = 0.0015<br>P = 0.0157<br>P < 0.0001
S5A	ANOVA Two Factor	$F_{(2, 12)}$=3.779 (I)<br>$F_{(1, 12)}$=111.6 (T)<br>$F_{(2, 12)}$=10.34 (G)	P = 0.0534<br>P < 0.0001<br>P = 0.0025
S5B	ANOVA Two Factor	$F_{(2, 12)}$=96.71 (I)<br>$F_{(1, 12)}$=63.53(T)<br>$F_{(2, 12)}$=147.0 (G)	P < 0.0001<br>P < 0.0001<br>P < 0.0001
S5C	ANOVA Two Factor	$F_{(2, 12)}$=26.27 (I)<br>$F_{(1, 12)}$=2.096 (T)<br>$F_{(2, 12)}$=21.50 (G)	P < 0.0001<br>P = 0.1733<br>P = 0.0001
